# Galectin-3 Binding to α_5_β_1_ Integrin in Pore Suspended Biomembranes

**DOI:** 10.1021/acs.jpcb.2c05717

**Published:** 2022-11-22

**Authors:** Nirod Kumar Sarangi, Massiullah Shafaq-Zadah, Guilherme B. Berselli, Jack Robinson, Estelle Dransart, Aurélie Di Cicco, Daniel Lévy, Ludger Johannes, Tia E. Keyes

**Affiliations:** † School of Chemical Sciences and National Centre for Sensor Research, Dublin City University, DCU Glasnevin Campus, D09 V209Dublin 9, Ireland; ‡ Institut Curie, PSL Research University, U1143 INSERM, UMR3666 CNRS, Cellular and Chemical Biology Unit, 75248Paris Cedex 05, France; § Institut Curie, PSL Research University, UMR 168 CNRS, 75248Paris Cedex 05, France

## Abstract

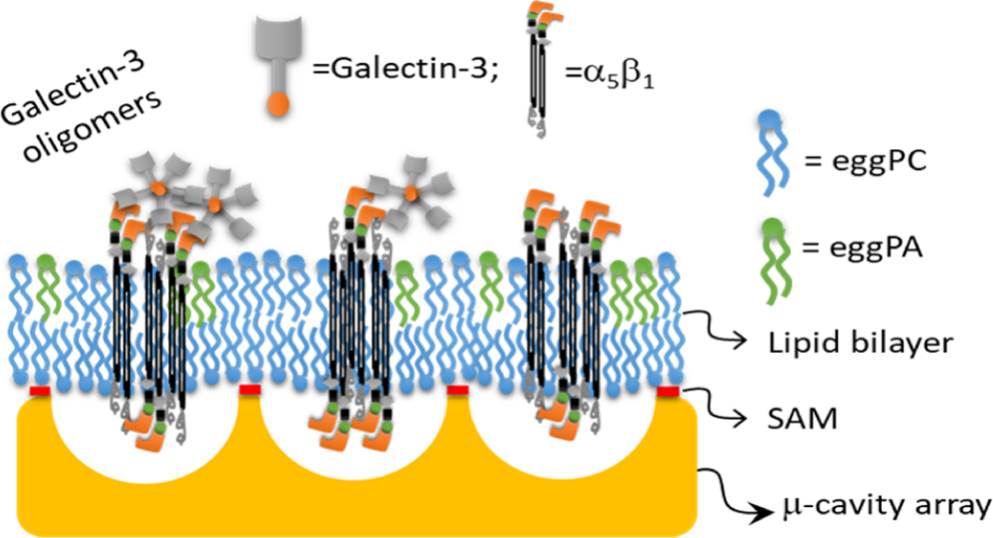

Galectin-3 (Gal3) is a β-galactoside binding lectin that mediates many physiological functions, including the binding of cells to the extracellular matrix for which the glycoprotein α_5_β_1_ integrin is of critical importance. The mechanisms by which Gal3 interacts with membranes have not been widely explored to date due to the complexity of cell membranes and the difficulty of integrin reconstitution within model membranes. Herein, to study their interaction, Gal3 and α_5_β_1_ were purified, and the latter reconstituted into pore-suspended lipid bilayers comprised eggPC:eggPA. Using electrochemical impedance and fluorescence lifetime correlation spectroscopy, we found that on incubation with low nanomolar concentrations of wild-type Gal3, the membrane’s admittance and fluidity, as well as integrin’s lateral diffusivity, were enhanced. These effects were diminished in the following conditions: (i) absence of integrin, (ii) presence of lactose as a competitive inhibitor of glycan–Gal3 interaction, and (iii) use of a Gal3 mutant that lacked the N-terminal oligomerization domain (Gal3ΔNter). These findings indicated that WTGal3 oligomerized on α_5_β_1_ integrin in a glycan-dependent manner and that the N-terminal domain interacted directly with membranes in a way that is yet to be fully understood. At concentrations above 10 nM of WTGal3, membrane capacitance started to decrease and very slowly diffusing molecular species appeared, which indicated the formation of protein clusters made from WTGal3−α_5_β_1_ integrin assemblies. Overall, our study demonstrates the capacity of WTGal3 to oligomerize in a cargo protein-dependent manner at low nanomolar concentrations. Of note, these WTGal3 oligomers appeared to have membrane active properties that could only be revealed using our sensitive methods. At slightly higher WTGal3 concentrations, the capacity to generate lateral assemblies between cargo proteins was observed. In cells, this could lead to the construction of tubular endocytic pits according to the glycolipid–lectin (GL–Lect) hypothesis or to the formation of galectin lattices, depending on cargo glycoprotein stability at the membrane, the local Gal3 concentration, or plasma membrane intrinsic parameters. The study also demonstrates the utility of microcavity array-suspended lipid bilayers to address the biophysics of transmembrane proteins.

## Introduction

Integrins are transmembrane heterodimeric proteins comprised of an α and a β subunit that mediate signals between the extra- and intracellular spaces via binding to different extracellular matrix ligands and numerous binding partners present at the cytosolic side of the plasma membrane.^1^ Twenty-four integrins are known in vertebrates that all have common structural motifs including in the α domain, a 7-bladed β propeller connected to a thigh and two calf domains. A metal ion-dependent adherent site is also conserved and is crucial for ligand binding.^2^ Integrins regulate various biological functions such as cell adhesion, migration, proliferation, differentiation, and spreading, and the remodeling of the extracellular matrix.^3^ This ability to regulate crosstalk between the cell and the surrounding environment positions integrins as key players in the process of tumor progression.^4^ Overexpression of integrins leads to therapy resistance, tumor reoccurrence, and survival issues. α_5_β_1_ integrin, also known as the fibronectin receptor, has been identified as a potential therapeutic target on certain solid tumors. For example, in breast cancer, it has been observed that the ligation of β_1_ integrins, such as α_5_β_1_ and α_2_β_1_, with extracellular matrix components significantly reduces drug-induced apoptosis from chemotherapeutic agents.^5^

Galectins are a family of 15 proteins that contain highly conserved carbohydrate-recognition domains (CRDs) that allow binding to glycosylated proteins and lipids at the surface of cells or in the extracellular matrix, including integrins. Galectins access the extracellular milieu by unconventional secretion.^6,7^ They have been classified into three subtypes, (i) prototype, (ii) tandem repeat, and (iii) chimera.^3,8−11^ A single CRD is found on prototype galectins, and two CRDs separated by a linker sequence on tandem repeat galectins.^12^

Galectin-3 (Gal3) is the sole representative of the chimera galectin subtype. This lectin has an important role, in part mediated by integrins, in physiological and pathological phenomena^13^ and is the most documented galectin family member. Gal3 is widely distributed in many tissues. It carries two specific domains, one CRD as for all galectin members, but also an intriguing N-terminal domain comprising proline/glycine/tyrosine-rich repeats. This N-terminal domain strongly determines the oligomerization capacity of Gal3 upon its binding to surface glycoproteins,^14,15^ possibly involving liquid–liquid phase separation.^16−18^ Gal3 is reported to stimulate neutrophil adhesion and migration, spreading of cancer cell lines and, furthermore, promotes focal adhesion turnover and fibronectin fibrillogenesis in tumor cells.^19−22^ Gal3 has been linked to various diseases such as cancer, type-1 diabetes, depression, and Alzheimer’s disease.^5,9,23−25^ Understanding and regulating the response of Gal3–integrin interactions is a critical biomedical challenge because lectin–integrin signaling is integral to many disease states.^26,27^

Galectins and notably Gal3 have been directly implicated in the regulation of the cell surface homeostasis of glycoproteins.^28^ They have been shown to favor the endocytic uptake of these glycoproteins, for example, α_5_β_1_ integrin^22,29^ via a mechanism that has been termed the glycolipid–lectin or GL–Lect hypothesis.^30^ According to this model, monomeric Gal3 in solution oligomerizes upon binding to cell surface glycoproteins such as integrins. Oligomeric Gal3 then acquires the capacity to interact with glycosylated lipids of the glycosphingolipid family in a way such as to drive the formation of tubular endocytic pits from which so-called clathrin-independent endocytic carriers form for the cellular uptake of the cargo glycoproteins. Initially described for Gal3 and the cellular uptake of CD44 and α_5_β_1_ integrin,^29^ the GL–Lect hypothesis has more recently also been documented for galectin-8 and another specific cargo protein, CD166.^31^ Although these findings are in apparent contradiction with earlier ones on galectins inducing lateral lattices that negatively affect endocytosis,^32^ we now envision a model in which galectin lattices and the GL–Lect mechanism cooperate to control the homeostasis of glycoproteins at the plasma membrane.^33^

In vitro studies at artificial membrane platforms, such as liposomes and supported lipid bilayers (SLBs), have been successfully applied to interrogate the interaction of membrane lipids as well as membrane-embedded receptors with external biomolecules such as proteins and peptides.^34−36^ Although important models, liposomes are somewhat limited in the approaches that can be applied to their analytical interrogation, for example, to study two-dimensional interfaces. Using fluorescence correlation spectroscopy (FCS) to analyze transmembrane proteins in giant unilamellar vesicles (GUVs) is possible because of their large size (10–50 μm). However, challenges with maintaining the focus of the confocal volume can arise due to the movement of the vesicle.^37^ Furthermore, as both leaflets of the liposomal bilayer are formed simultaneously, asymmetric leaflet composition is challenging to achieve with precision.^38,39^ While compositionally and analytically more versatile, SLBs suffer interference from the interfacial support (substrate effect) due to frictional/pinning on the fluidity and functionality of the bilayer and associated membrane proteins that can limit biomimicry. Tethered or cushioned membranes offer improved biomimicry by introducing an extra layer between the substrate and lower leaflet, through a covalently bound self-assembled monolayer or a physio-absorbed polymer cushion; yet, challenges around friction/lateral movement of proteins still remain.^35,40−43^ Nonetheless, the advantages of SLB-based approaches are their greater compositional control and versatility in terms of experimental interrogation compared with liposomes.^44−46^ Particularly, when the solid support is conducting, this can be used as an electrode enabling the electrochemical study of lipid–peptide/protein interactions.^47−50^

Alternative approaches have emerged recently, in which membranes supported over buffer-filled periodic pore structures are assembled, which improves membrane fluidity, while maintaining stability. Most importantly, in the case of buffer-filled pore-supported bilayers, they offer the advantage of a relatively deep aqueous reservoir in contact with the proximal leaflet that SLBs lack.^51−55^ We recently exploited microcavity array-suspended lipid bilayers (MSLBs) formed at cavity array polydimethylsiloxane (PDMS) and gold electrodes to study receptor-mediated interactions and detection of peripheral proteins such as the B-subunit of cholera toxin,^56^ hemagglutinin A1,^57^ annexin V,^58^ and small-molecule drug permeability,^59−62^ across varied lipid membrane compositions, and have demonstrated that they offer the fluidity of liposome/proteoliposomes with the addressability of SLBs. Here, we have applied them to a biophysical study of α_5_β_1_ integrin–Gal3 interactions.

## Materials and Methods

### Equipment and Reagents

Rat livers (Charles River), functionalized wheat germ agglutinin (WGA) agarose resin (Sigma-Aldrich, ref. 61768-5 mL), NHS-activated agarose column functionalized with FN-III_9–10_ (GE Healthcare, NHS-HiTrap ref. 17071701), pre-casted 4–15% polyacrylamide gels (Bio-Rad), 300–400 mesh carbon-coated copper grids for electron microscopy (EM) (Delta Microscopy, ref. DG400-Cu), 4-(2-hydroxyethyl)-1-piperazineethanesulfonic acid (HEPES) 1 M, pH 7–7.4 (Sigma-Aldrich, ref. H0887), MgCl_2_, CaCl_2_, Triton X-100 (Anatrace, ref. T1001-500 mL), *n*-dodecyl β-d-maltoside (DDM) (Anatrace, ref. D310-25GM), protease inhibitors (Sigma-Aldrich, ref. P8849), Pefabloc (Sigma-Aldrich, ref. 76307), *N*-acetylglucosamine (GlcNac, Sigma-Aldrich, ref. A8625-5G), sucrose, ethylenediaminetetraacetic acid (EDTA) pH 8, chicken egg phosphatidylcholine (Avanti Polar, ref. 840051C), chicken egg l-α-phosphatidic acid (Avanti Polar, ref. 840101C), rhodamine B 1,2-dihexadécanoyl-*sn*-glycéro-3-phosphoéthanolamine (Thermo Fisher, ref. L1392), HisPur cobalt resin (Thermo Fisher, ref. 89965), NHS-ATTO488, ATTO655 DOPE, ATTO532 DOPE (ATTO-TEC GmbH), NHS-Alexa647 (Invitrogen), non-reducing sodium dodecyl sulfate (SDS)-loading sample buffer, hamster anti-rat β_1_ integrin (BioLegend, ref. 102202) primary antibody, HRP-coupled secondary anti-hamster antibody, and ECL. Bio-Beads SM2 adsorbent media (Bio-Rad, ref. 152-3920), Bio-Rad ChemiDoc for protein detection (chemiluminescence).

### Purification of α_5_β_1_-Integrin from Rat Livers

α_5_β_1_ integrin was solubilized and purified as described earlier.^63,100^ Briefly, 4 rat livers (about 65 g in total) were cut in small pieces and incubated with lysis buffer [20 mM HEPES, 2 mM MgCl_2_, 2 mM CaCl_2_, 2% (v/v) Triton X-100, protease inhibitor cocktail 1/250 dilution, and Pefabloc 1/1000 dilution, pH 7.4], such that the liver tissue weight represents 25% (w/v) of the total final volume of extraction. Tissues were 2 times homogenized using an ultra-Turrax homogenizer and incubated for 60 min at 4 °C on orbital shaker. The extract was centrifuged at 75,000*×g* for 30 min at 4 °C. The supernatant was then centrifuged again using the same settings. The final lysate supernatant was clarified by filtration through a Whatman paper and loaded on two 25 mL WGA-affinity columns connected in series, at a flow rate of 0.8 mL/min. Columns were washed with washing buffer [20 mM HEPES, 150 mM NaCl, 2 mM MnCl_2_, 0.2% (v/v) Triton X-100, protease inhibitor cocktail 1/250 dilution, Pefabloc 1/1000 dilution, pH 7.4], at a flow rate of 2 mL/min. Glycosylated proteins were finally eluted with washing buffer supplemented with 300 mM GlcNAc. Protein-enriched fractions were pooled and loaded on a 10 mL FN-III_9–10_-functionalized column at a flow rate of 0.08 mL/min, to specifically bind α_5_β_1_ integrin, using an FPLC purifier system. Unbound material was then removed with washing buffer at a flow rate of 0.2 mL/min. α_5_β_1_ integrin was finally eluted with elution buffer [20 mM HEPES, 150 mM NaCl, 10 mM EDTA, 0.2% (v/v) Triton X-100, Pefabloc 1/1000, pH 7.4], at a flow rate of 0.08 mL/min.

For EM and negative staining characterization of purified α_5_β_1_ integrin, protein extraction from rat livers was performed using DDM instead of Triton X-100.

Protein purity was determined by SDS-PAGE analysis. Integrin-enriched fractions were then pooled and final concentration determined by the Bradford colorimetric assay. Integrin was snap-frozen and stored at −80 °C.

### Negative Staining and Electron Microscopy

α_5_β_1_ integrin reconstitution was performed at a lipid/protein ratio (LPR) of 2700 (molmol). 200 μg of eggPC:eggPA (9:1, mol/mol) solubilized in chloroform were mixed and excess of organic solvent evaporated under a nitrogen atmosphere. Lipidic films were dried under vacuum at room temperature for 2 h, and then resuspended in 150 μL of 20 mM HEPES, 150 mM NaCl pH 7.4. Lipids were then solubilized by adding 0.25% (v/v) Triton X-100 and incubated for 10 min at 21 °C under gentle stirring. 20 μg of purified α_5_β_1_ integrin was added to the solubilized lipids, the Triton X-100 concentration was adjusted to 0.5% (v/v) in a 200 μL final reconstitution volume and incubated for 10 min at 21 °C under gentle stirring. The detergent was eliminated by three consecutive additions of Bio-Beads SM2 (prepared according to manufacturer’s instruction), 2 times with 10 mg, and 1 time with 20 mg, after 2, 1 ,and 1 h of time intervals, respectively. Lipid and α_5_β_1_ integrin final concentrations in small unilamellar vesicles (SUVs) were 1 and 0.1 mg/mL, respectively. This led to complete detergent removal and the formation of unilamellar vesicles.^64^ SUVs were transferred to a new tube and stored at 4 °C until use. The size of the integrin reconstituted SUVs was estimated from dynamic light scattering (DLS) using a Malvern, Zetasizer (Figure S1, Supporting Information).

### Characterization of α_5_β_1_ Integrin Incorporation into SUVs by Floatation in a Sucrose Gradient

For SUV visualization on sucrose gradient, 0.5% (w/w) of rhodamine DHPE was added to the lipid composition of α_5_β_1_ integrin/SUVs, prepared, as described above. In a cold room, 100 μL of α_5_β_1_ integrin/SUVs were diluted to a final volume of 575 μL in 20 mM HEPES, 150 mM NaCl, 30% (w/v) sucrose, pH 7.4, and placed at the bottom of a 2.5 mL polypropylene ultra-centrifuge tube. Sucrose gradient was then built by gently pouring, from the bottom to the top of the tube, 575 μL of 20, 10, 5, and 0% (w/w) sucrose solutions prepared in 20 mM HEPES and 150 mM NaCl pH 7.4 buffer. The sample was centrifuged at 100,000×*g* (Beckman TLS55 rotor) for 16 h at 4 °C. Samples were collected from each sucrose density, denatured in non-reducing SDS-sample loading buffer, boiled for 5 min, and loaded on a 4–15% pre-casted polyacrylamide gel. Proteins were then transferred on the nitrocellulose membrane, and the presence of α_5_β_1_ integrin in all fractions was immuno-detected by incubation with primary hamster anti-rat β_1_ integrin antibodies, followed by secondary HRP-coupled anti-hamster antibody.

### Characterization of α_5_β_1_/SUV Morphology by Cryo-EM

4 μL of α_5_β_1_ proteoliposomes were loaded on a glow-discharged lacey carbon 300 mesh grids. Blotting was carried out on the opposite side from the liquid drop and samples were plunge frozen in liquid ethane (EMGP, Leica, Germany). Cryo-EM images were acquired with a Tecnai G2 (Thermo Fisher, USA) Lab6 microscope operated at 200 kV and equipped with a 4k × 4k CMOS camera (F416, TVIPS). Image acquisition was performed under low dose conditions of 10 e^–^/Å^2^ at a magnification of 50,000 with a pixel size of 2.14 Å.

### Characterization of α_5_β_1_ Orientation after Reconstitution in SUVs

5 μL of α_5_β_1_/SUVs were diluted with 5 μL of 20 mM HEPES,150 mM NaCl pH 7.4 buffer, and incubated under agitation for 30 min at 20 °C with 10 μL of 200 μg/mL trypsin solution, either complemented with 0.5% (v/v) Triton X-100 (total integrin digestion) or not (surface-exposed integrin digestion). Trypsin was inactivated with 10 μL of non-reducing SDS-sample loading buffer and boiled for 5 min at 95 °C. Samples were loaded on 4–15% pre-casted polyacrylamide gels and proteins were then transferred onto nitrocellulose membranes. The presence of α_5_β_1_ integrin was immuno-detected by incubation with primary hamster anti-rat β_1_ integrin antibody, followed by secondary HRP-coupled anti-hamster antibody.

### Functionality of α_5_β_1_ Integrin after Reconstitution in SUVs

α_5_β_1_ integrin functionality, once incorporated in SUVs, was assessed by its capacity to bind fibronectin. 20 μL of α_5_β_1_/SUVs (2 μg of protein and 20 μg of lipids) were incubated for 30 min at 21 °C on a rotating wheel either with 2 mM MgCl_2_ or MnCl_2_ in 100 μL of 20 mM HEPES,150 mM NaCl, pH 7.4 buffer. Histidine-tagged FN-III_9–10_ fibronectin fragment was added at a 10 μM final concentration for 90 min at 21 °C under rotation. Reactions were loaded on 30 μL bed volume of cobalt-coated beads and incubated for one additional hour at 4 °C on a rotating wheel. Unbound materials were then removed, and beads washed with 20 mM HEPES, 150 mM NaCl, pH 7.4 buffer, supplemented or not with 2 mM of MgCl_2_ or MnCl_2_. Proteins were eluted from the beads with 30 μL of non-reducing SDS-loading sample buffer and boiled 5 min at 95 °C. Samples were loaded on a 4–15% pre-casted polyacrylamide gel, and proteins were then transferred onto the nitrocellulose membrane. The presence of α_5_β_1_ integrin was immuno-detected by incubation with primary hamster anti-rat β_1_ integrin antibody, followed by secondary HRP-coupled anti-hamster antibody.

### Labeling of α_5_β_1_ Integrin with ATTO488 Fluorophore

Triton X-100-purified α_5_β_1_ integrin [20 mM HEPES, 150 mM NaCl, 0.2% (v/v) Triton X-100] was incubated with a 10 molar excess of NHS-ATTO488 for 2 h at 21 °C under gentle agitation. The reaction was quenched by the addition of 20 mM Tris pH 7.4 for 20 min at 21 °C. Excess of NHS-ATTO488 was then removed using 40 kDa cutoff desalting spin-columns equilibrated with 20 mM HEPES, 150 mM NaCl, 0.2% (v/v) Triton X-100, according to the manufacturer’s instructions. Final protein concentration was assessed by a BCA colorimetric assay.

### Labeling of Wild-Type Gal3 and Gal3ΔNter with Alexa647

Wild-Type Gal3 (WTGal3) or Gal3ΔNter at a final concentration of 2 mg/mL were incubated for 2 h at 21 °C under gentle shaking in phosphate-buffered saline (PBS) with 4 molar excess of NHS-Alexa647 dye, in the presence of 10 mM of β-lactose. Reactions were quenched with 20 mM Tris final concentration for 20 min at 21 °C. Unreacted dye was cleared using 7 kDa desalting columns equilibrated with PBS, according to manufacturer’s instructions. Protein concentration and labeling efficiency were determined by measuring OD_280_ and OD_647_, respectively.

### Fabrication of Microcavity Array Gold and PDMS Substrate

Microcavity SLBs were assembled across periodic pore arrays prepared in PDMS for fluorescence correlation studies, or across gold pore array electrodes for electrochemical studies where the porous arrays were prepared by polystyrene sphere-templating methods as previously described.^56,57,61,65^ Briefly, to obtain hexagonally close-packed microcavity array on gold a monolayer of 1 μm polystyrene microspheres was cast using a gravity-assisted method onto pre-cut rectangles of gold-coated silicon wafers (Figure S2, Supporting Information). Gold was electrodeposited onto the interstitial surface between the polystyrene microspheres by applying a reduction potential (−0.6 V, Ag/AgCl) to the gold array in the presence of a cyanide-free gold solution. The electrodeposition was controlled by monitoring the evolution of the current at the gold array until the current reached a minimum value corresponding to the closer distance between the spheres, indicating that the electrodeposition of gold has reached the hemisphere of polystyrene (Figure S2, Supporting Information). After the gold electrodeposition, the arrays were electrochemically cleaned using cyclic voltammetry in 50 mM sulfuric acid for 3 cycles within −0.2 to 1.6 V potential (vs Ag/AgCl) (Figure S2, Supporting Information) and rinsed with deionized water, ethanol, and dried gently under nitrogen flow. The top surface of the gold microcavity arrays was then selectively functionalized with a self-assembled monolayer (SAM) of 6-mercaptohexanol (1 mM) for at least 24 h in ethanol, before removal of the templating spheres, which were subsequently washed out of the array with tetrahydrofuran (THF).^56,62^ An electroactive area of ∼5 cm^2^ was determined for electrodes used in this study for MSLB preparation and subsequent electrochemical experiments (see the Supporting Information).

The PDMS microcavity arrays were prepared by drop-casting 50 μL of ethanol containing 0.1% of 4.61 μm diameter polystyrene spheres (Bangs Laboratories) onto a 1 cm × 1 cm hand cleaved mica sheet, which was glued to a glass coverslip. After ethanol evaporation, PDMS was poured onto the polystyrene microsphere arrays and cured at 90 °C for 1 h. Microcavity arrays were formed after removing the inserted polystyrene microspheres by sonicating the PDMS substrate in THF for 15 min. The substrates were then left to dry overnight. Prior to lipid bilayer formation, the substrates were cleaned using oxygen plasma for 5 min and microcavities were buffer filled before lipid monolayer deposition by sonicating the PDMS substrate in PBS buffer (pH 7.4) for 1 h.

### Fabrication of Pore-Suspended Lipid Bilayers

Pore-suspended lipid bilayers were prepared according to a two-step process described previously for a range of bilayer compositions.^55,57−62,65^ The proximal leaflet of eggPC monolayers was transferred by the Langmuir–Blodgett (LB) technique. Briefly, approximately 50 μL of eggPC (1 mg/mL in chloroform) were deposited onto the air/water interface of LB trough (NIMA 102D) and the solvent was allowed to evaporate for 15 min. The resulting lipid monolayer at the air/water interface was compressed 4 times to a surface pressure of 33 mN/m at 25 mm/min. Then, the aqueous-filled microcavity arrays were immersed into the LB trough until all of the cavities were submerged completely into the subphase. The micro-cavity array was withdrawn from the trough at a rate of 5 mm/min while the surface pressure of the lipids was maintained at 32 mN/m to ensure an adequate transfer of the eggPC monolayer. To assemble the upper leaflet of the bilayer, liposome (with or without α_5_β_1_ integrin) fusion (Figure S2, Supporting Information) was carried out using SUVs prepared as described above, comprised of eggPC:eggPA (90:10). Where integrin was reconstituted into the MSLB, according to the procedure above, a proteoliposome was prepared at a LPR of 2700 and fused to the LB prepared monolayer. Following LB transfer, the fusion processes were carried out within a sealed microfluidic chamber for PDMS arrays. For gold, 150 μL liposome/proteoliposome solution was introduced to the monolayer. The bilayers are not exposed to air/allowed to dry, whether during the liposome wash step or throughout measurement. The resulting PDMS or gold MSLBs were confirmed formed by the fluorescent lifetime imaging (FLIM), FCS, or by electrochemical methods, respectively.

### Fluorescence Lifetime Correlation Spectroscopy

Fluorescence lifetime imaging and correlation spectroscopy measurements were performed using a Microtime 200 system (PicoQuant GmBH, Germany) integrated with a FCS module, dual SPD detection unit, time-correlated single photon counting, on an inverted Olympus X1-71 microscope with an Olympus UPlanSApo 60×/1.2 water immersion objective. A single mode optical fiber guided the lasers to the main unit and provided a homogeneous Gaussian profile excitation beam. The lasers were pulsed at 20 MHz, corresponding to an interval of 50 ns. The emitted fluorescence was collected through the microscope objective. A dichroic mirror z532/635rpc blocked the backscattered light, and corresponding filters were used to clean up the signal. A 50 μm pinhole was set to confine the volume of excitation and detection of fluorescence intensity that originated from the fluorescently labeled protein in the axial direction. Fluorescence was detected using a single photon avalanche diode from MPD (PicoQuant). Before the FCLS measurement, backscattered images of the substrate (images were collected using an OD3 density filter) followed by fluorescent lifetime images were acquired to ensure the optimal positioning of the buffer-filled cavities where the bilayers are spanned. Fluorescence lifetime correlation spectroscopy (FLCS) analyzes the time-dependent fluctuations of the fluorescence intensity ∂*I*(*t*) recorded over 30 s and analyzed by an autocorrelation function (ACF), *G*(τ) = (⟨∂*I*(*t*)⟩·⟨∂*I*(*t* + τ)⟩)/⟨∂*I*(*t*)⟩^2^, where ⟨⟩ denotes the time average, and ⟨∂*I*(*t*)⟩ and ⟨∂*I*(*t* + τ)⟩ are the fluorescent intensity fluctuations around the mean value at time *t* and *t* + τ, respectively. The FLCS autocorrelation data fit to a 2D diffusion model equation defined in eq 1
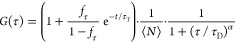
1where, ⟨*N*⟩ is the average number of diffusing fluorescence particles in the observation volume, *f*_τ_ and τ_T_ are the fraction and the decay time of the triplet state, τ_D_ is the transit time, and α is the anomaly coefficient. From the fitting, the τ_D_ values were evaluated and accordingly, the diffusion coefficient was obtained from eq 2
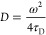
2where, ω is the observation beam waist diameter, typically obtained from the calibration of a standard dye diffusing in 3D with a known diffusion coefficient value. Point FLCS measurements were then recorded at the center of cavity for a duration of 30 s and an average of 20 cavities were studied for each sample for every FLCS measurements. All FLCS experiments were carried out in triplicate at 20 ± 1 °C.

### Electrochemical Impedance Spectroscopy

Electrochemical impedance spectroscopy (EIS) measurement was performed with a CHI760e (CH Instruments, USA). A standard 3-electrode cell was employed for all measurements, comprised of a gold microcavity array covered by the lipid bilayer as a working electrode, an Ag/AgCl (1 M KCl) reference, and a coiled platinum wire counter electrode. The EIS data were recorded across a frequency range of 0.05 to 10^5^ Hz with an AC modulation amplitude of 10 mV at a DC potential bias of 0 V (vs Ag/AgCl). All measurements were carried out in a glass cell (approximate volume of 4 mL of 0.01 M PBS). The EIS of the aqueous-filled microcavity array coated with the lipid bilayer composition alone was measured initially before the addition of galectin to ensure signal stability. The non-Faradaic EIS signal from the integrin-reconstituted MSLBs was evaluated for stability, and it was found that when placed in contact with the buffer, an initial fluctuation of resistance occurred that stabilized within an hour and then remained unchanged over a prolonged 24 h window, which was well beyond our experimental time window (5–6 h) for WTGal3/Gal3ΔNter binding studies. Initially, for integrin-reconstituted MSLBs, a time lag of 90–120 min was allowed to ensure that the membrane had equilibrated in the electrochemical cell (no fluctuation of EIS, i.e., the membrane impedance is unchanged) before proteins were titrated. For each protein aliquot, the designated concentration of proteins was added to the electrochemical cell and an equilibration binding time of 30 min was maintained. This window was confirmed to be sufficient for protein binding to the membrane, as beyond this time, protein binding elicited no further change to a membrane impedance signal. The proteins (WTGal3 and Gal3ΔNter) were initially prepared as a stock solution in buffer and this was aliquoted into the electrochemical cell to achieve the required final concentration and mixed thoroughly. The volume added to the cell never exceeds 200 μL. All measurements were carried out at room temperature (22 ± 1 °C). The impedance of the MSLBs for each protein type, as well as their temporal stability was assessed in triplicate. The measured data were analyzed using Z-View software (Scribner Associates, v3.4e) by fitting the equivalent circuit model (ECM), as shown in Figure 2C. The best fit using the ECM circuit was assessed from both visual inspections of the fit residuals and χ^2^, typically ∼0.001.

## Results and Discussion

### Purification, Characterization, and Reconstitution of α_5_β_1_ Integrin within Small Unilamellar Vesicles

**Figure 1:**
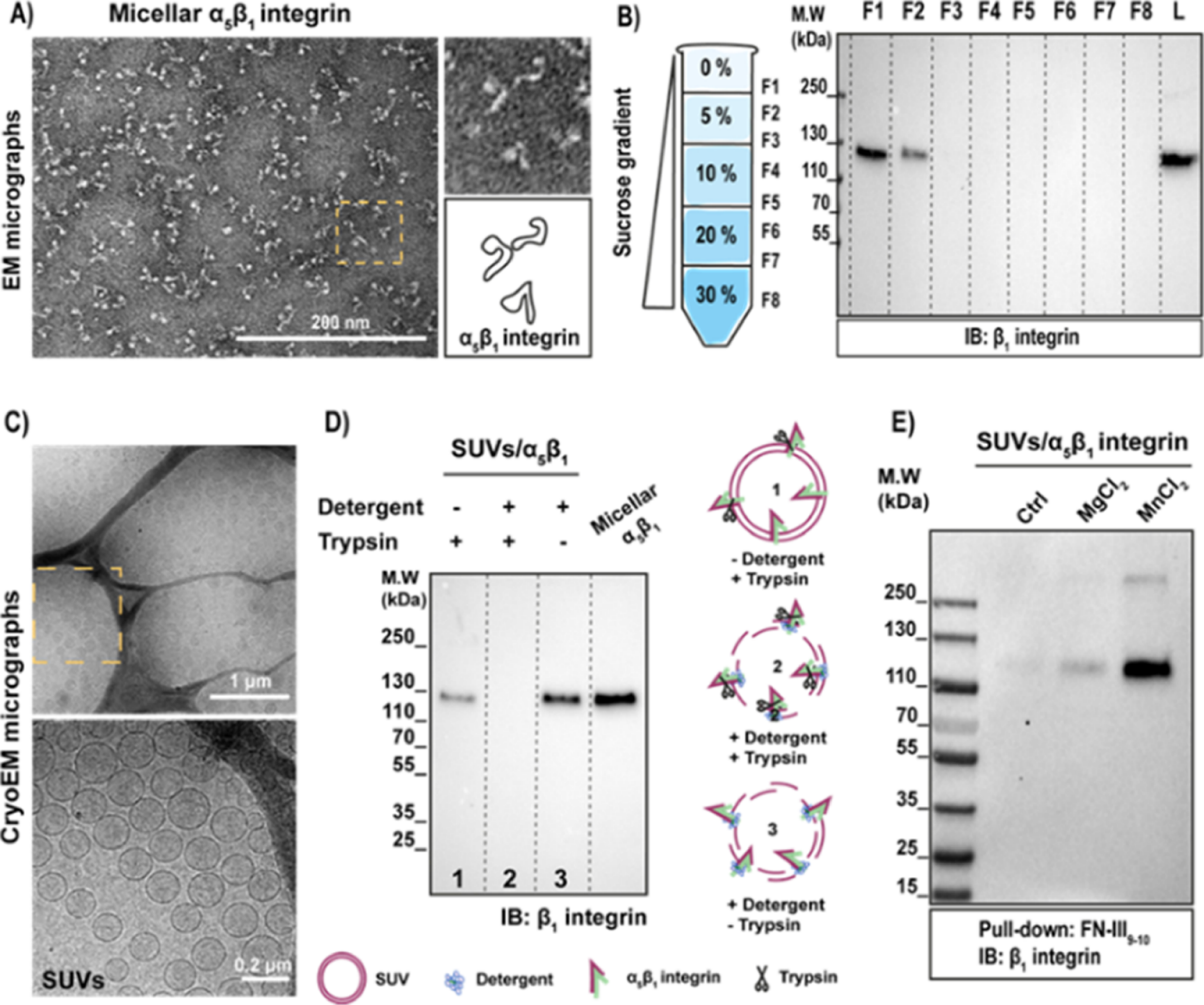
α_5_β_1_ integrin purification and reconstitution into SUVs. (A) Qualitative visualization of α_5_β_1_ integrin particles by EM. Negative staining images of α_5_β_1_ integrin solubilized in DDM. The inset shows a zoomed image of integrin particles, and an illustration of individual integrins in the bent-closed conformational state. (B) Characterization on sucrose gradients of α_5_β_1_ integrin incorporation into vesicles. Gradient fractions F1 to F8 (right cartoon illustration) were collected and submitted to anti-β_1_ integrin immunoblotting. β_1_ integrin was expectedly detected at 120 kDa in F1 and F2 fractions. L represents total proteoliposome input. (C) Homogeneity of the proteoliposomes as visualized by cryoEM. Inset shows a magnification view. The proteoliposomes were homogenous in size, with a mean diameter of 150 nm. (D) Analysis of α_5_β_1_ integrin orientation within SUVs. Proteoliposomes were subjected or not to trypsin digestion in the presence or absence of Triton X-100. In the absence of detergent (lane 1), the immunoreactive band corresponds to β_1_ integrin molecules for which the large extracellular domain was oriented into the liposomal lumen and thereby protected from the protease. In the presence of trypsin and detergent (lane 2), no β_1_ integrin band was detected, since the enzyme now had full access to the whole protein. In the presence of the detergent alone (lane 3), the detected band corresponds to the total amount of β_1_ integrin. This allowed us to estimate the percentage of correctly oriented α_5_β_1_ integrin, which was around 50%. Micellar α_5_β_1_ integrin was used as a control. The cartoon illustration summarizes the different conditions. (E) Assessment of the functionality of liposomal α_5_β_1_ integrin. To confirm the capacity of α_5_β_1_ integrin to become activated. Proteoliposomes were pre-incubated or not (Ctrl) with the indicated activating metal ion salts (MgCl_2_ or MnCl_2_) and then submitted to fibronectin FN-III_9–10_ pull down. As expected, β_1_ integrin was pulled down in the presence of magnesium (MgCl_2_) and to a greater extent in the presence of manganese (MnCl_2_), demonstrating that the SUV-reconstituted integrins were functional.

α_5_β_1_ integrin was solubilized in DDM detergent and purified from the rat liver, as described in the Materials and Methods section. We have used EM in the negative staining mode to qualitatively validate the integrity of the DDM-micellar protein particles at the structural level. Purified micellar α_5_β_1_ integrin clearly appeared as individual particles on the EM grids that presented the typical shape of integrin heterodimers, including a globular headpiece and thinner leg parts (Figure 1A). Many of these integrin particles were in the bent-closed inactive conformation, 10–15 nm in length and well described for their low affinity for fibronectin (Figure 1A, inset, illustration). This was fully consistent with our elution strategy, where the integrin was initially activated to efficiently bind a FNIII_9–10_-functionalized column, while the subsequent release was achieved by shifting the protein to the inactive conformation, which led to its elution from the column in the bent-closed conformation.

We then successfully incorporated the purified micellar-integrin (Triton X-100 micelles) into SUVs at a low protein density with a LPR of 2700 (mol/mol). The rate of integrin incorporation in SUVs was assessed by loading the reconstituted proteoliposomes onto sucrose gradients, followed by immunodetection of the protein in each fraction. Interestingly, integrins were only detected at the middle and the top fractions of the 5% sucrose gradient (Figure 1B). This suggested a homogenous distribution of the protein among the proteoliposome populations and a low density of protein, as expected from the LPR that was used. Moreover, the absence of integrin in the fraction corresponding to the highest concentration of sucrose (here 30%) indicated that no protein aggregation occurred during the reconstitution step.

Cryo-EM analysis was performed to assess both the morphology and size of the α_5_β_1_ integrin proteoliposomes. As shown on the micrographs, these liposomes appeared as homogenous unilamellar and spherical vesicles, with a mean diameter of 150 nm (Figure 1C, inset), suitable for subsequent reconstitution onto the MSLBs.

The orientation of α_5_β_1_ integrin after reconstitution in SUV was then assessed. It is indeed critical to have a substantial fraction of integrin molecules that are correctly oriented within the proteoliposomes with their cytosolic domain facing into the lumen. The trypsin-accessibility/protection assay was used to address this point. Proteoliposomes were incubated with trypsin in the absence of Triton X-100 for the immunodetection of the luminally orientated integrin fraction [Figure 1D, lane 1, and illustration (top right panel)], or in the presence of Triton X-100 without trypsin [Figure 1D, lane 3, and summarized in the illustration (bottom right panel)] for evaluation of the total amount of incorporated integrin to *in fine* estimate the proportion of correctly oriented integrin. The efficiency of enzymatic digestion was confirmed by the absence of β_1_ integrin immunodetection [Figure 1D, lane 2, and illustration (middle right panel)] when SUVs were disrupted by Triton X-100 incubation before trypsin treatment, thus exposing the total pool of integrin to the enzyme. Importantly, integrins were symmetrically oriented in the membrane with 50% of the protein pointing outward from the vesicles.

We then aimed to confirm that our reconstituted α_5_β_1_ integrin was functional. For this, we have assessed its capacity to become activated, either using MgCl_2_ or MnCl_2_ preincubation for integrin priming, followed by pull-down on fibronectin (FNIII_9–10_) fragment-coated beads. Immunodetection of β_1_ integrin in the presence of MgCl_2_, and to a greater extent with MnCl_2_ (Figure 1E), confirmed that even in the liposomal environment of SUVs, α_5_β_1_ integrin retained its capacity to become activated and to interact efficiently with its natural ligand, fibronectin.

### Characterization of Proteoliposomes Using DLS and Fluorescence Life-Time Cross-Correlation Spectroscopy

DLS was used to follow each of the steps of proteoliposome preparation. The initial eggPC:eggPA liposomes resuspended in HEPES buffer formed vesicles of narrow size distribution with a mean diameter of 500 nm (Figure S1A, Supporting Information). Detergent disruption was confirmed from the change in the size distribution of liposomes (Figure S1B, Supporting Information). Following detergent removal, the reformed proteoliposomes containing α_5_β_1_ integrin showed a homogeneous diameter of 120 nm (Figure S1C, Supporting Information). Additionally, using fluorescence life-time cross-correlation spectroscopy, the diffusion of labeled α_5_β_1_ integrin-ATTO488 and DOPE-ATTO655 in proteoliposomal membranes was evaluated following simultaneous excitation of both fluorophores (Figure S1D, Supporting Information). The ACF showed that α_5_β_1_ integrin and DOPE co-diffused, confirming that both were indeed reconstituted into the same proteoliposomes.

### Characterization of Integrin-Reconstituted MSLB over Aqueous-Filled Gold Substrate

**Figure 2:**
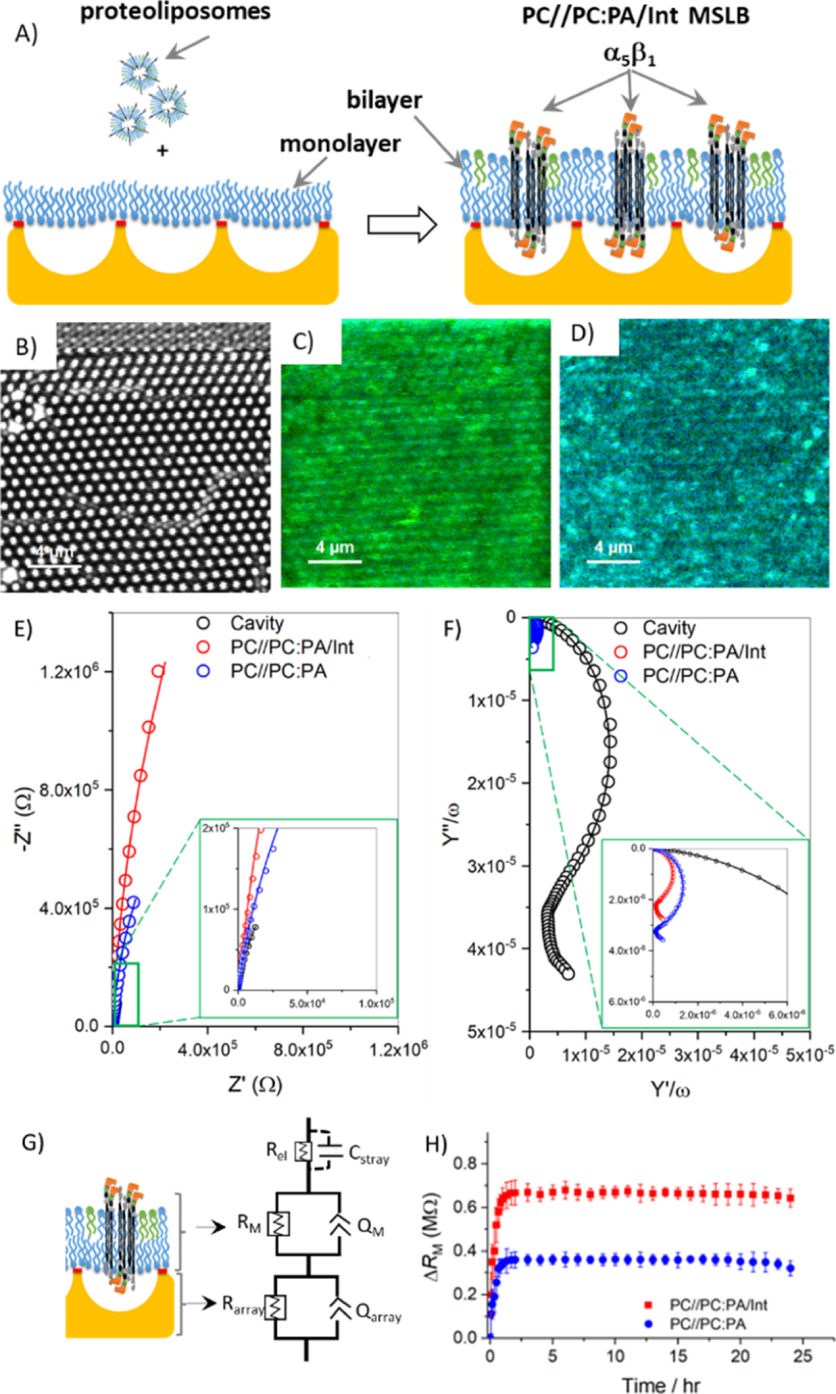
(A) Schematic illustration of proteoliposome fusion over a lipid monolayer covered gold microcavity array (not to scale) yielding PC//PC:PA/Int MSLBs. Reflectance image (B), FLIM images obtained from the lower PC leaflet doped with 0.03 mol% ATTO647-PE (C), andfrom ATTO488 α_5_β_1_ integrin (D). Non-Faradaic Nyquist plot (E) and frequency–normalized complex capacitance plot (F) of the cavity array alone (black), of PC//PC:PA (blue) and of PC//PC:PA/Int MSLBs (red). In panels E and F, zoomed-in areas are shown in insets as indicated by the green boxes. (G) Schematics of MSLB spanned over a microcavity (not to scale) and the associated ECM used to fit EIS data. In the ECM, *R*_el_, and *C*_stray_ represent, respectively, solution electrolyte resistance and stray capacitance, *R*_M_ and *Q*_M_ represent, respectively, membrane resistance and CPE, and *R*_array_ and *Q*_array_ are the, respectively, microcavity array resistance and CPE. The corresponding fits to the ECM are shown as solid lines in panels E and F. (H) Relative change in membrane resistance to show the stability of PC//PC:PA membranes without (blue) and with (red) the presence of α_5_β_1_ integrin versus time monitored for more than 24 h. The EIS recording at an initial time window of 0–1.5 h shows an increase in membrane resistance, which saturates and remains stable for more than 24 h. EIS measurements were performed in PBS buffer within the frequency ranges between 0.05 and 10^5^ Hz at 0 V DC bias potential vs Ag/AgCl (1 M KCl) with an AC amplitude of 10 mV at 22 ± 1 °C. A three-electrode setup where gold cavity/MSLB, Ag/AgCl (1 M KCl), and Pt wire served as working, reference, and counter electrodes, respectively.

Gold microcavity-suspended lipid bilayers (MSLB, pore diameter, 1 μm) comprising of eggPC (PC) at the proximal leaflet, eggPC:eggPA (PC:PA) at the distal leaflet, and bilayer-spanning integrin were prepared via LB transfer of a PC monolayer followed by fusion of proteoliposomes, as shown schematically in Figures 2A and S2A (Supporting Information). These MSLBs are hereafter called PC//PC:PA/Int. The proteoliposomes comprised of α_5_β_1_ integrin reconstituted into PC:PA (90:10) SUVs (∼120 nm diameter) at a LPR of 2700. Fluorescence lifetime imaging was used to characterize the PC//PC:PA/Int MSLB assembly at the gold microcavity array. To facilitate this, the lower PC leaflet, transferred during the LB deposition was doped with ATTO647-PE (0.03 mol %) and α_5_β_1_ integrin was fluorescently labeled with ATTO488.

A representative reflectance image of PC//PC:PA/Int MSLB from a gold microcavity array is shown in Figure 2B. The white circular features show reflected light from the buffer-filled cavities, replicated across the surface. Reflectance imaging indicates that at the gold array, all the cavities are buffer filled. Figure 2C,D shows representative FLIM images obtained from the fluorescently labeled lower leaflet and the integrin, respectively, confirming that a bilayer had formed and that the integrin was reconstituted into the bilayer. The PC//PC:PA:Int bilayer was also characterized by atomic force microscopy (AFM) (Figure S3, Supporting Information). To further confirm bilayer formation from the LB/liposome fusion process, we separately labeled each monolayer of PC//PC:PA membranes that were assembled over gold microcavities. The lower PC leaflet was labeled with ATTO532-PE, and the upper PC:PA leaflet with ATTO647-PE. The corresponding reflectance and FLIM images show, as expected, a continuous fluorescence signal from each leaflet across the entire array surface (Figure S4, Supporting Information).

Exploiting the gold microcavity array as a working electrode, we then characterized the PC//PC:PA/Int MSLB using EIS. Figure 2E shows representative non-Faradaic Nyquist plots obtained from EIS measurements before (black) and after PC//PC:PA/Int (red) spanned over SAM-modified cavity arrays. The corresponding frequency–normalized complex capacitance plots are shown in Figure 2F. The non-Faradaic Nyquist trace shows the sum of real (*Z*′) and imaginary (−*Z*″) components of the complex impedance, which reflect any changes to the ion-transfer process across electrode/electrolyte interface. For example, when the Nyquist trace shifts toward the *x*-axis (*Z*′), the impedance is decreased, or admittance (ion transport) is increased. Similarly, a shift toward the *y*-axis (−*Z*″) implies that the impedance is increased, and admittance decreased. As expected, on PC//PC:PA/Int (red) bilayer formation, the impedance was increased greatly compared to the SAM-functionalized cavity array (black) support (Figure 2E, inset). In addition, the electrode capacitive properties on the assembly of PC//PC:PA/Int can be visualized from the angular frequency–normalized complex capacitance plot (*Y*″/ω vs *Y*′/ω), as shown in Figure 2F. The semicircle plot intercept in *Y*″/ω at ∼40 × 10^–6^ F for a SAM-modified cavity (black) was significantly reduced to 2.1 × 10^–6^ F for α_5_β_1_ integrin-containing membranes (Figure 2F and inset). As a control, the pristine PC//PC:PA (blue) membrane without α_5_β_1_ integrin is also included in panels E and F. When compared to the α_5_β_1_ integrin containing membrane, the pristine PC//PC:PA membrane (red) showed a lowering in resistance (blue, Figure 2E) and a modest increase in capacitance (3.2 × 10^–6^ F). Because capacitance is inversely proportional to the thickness of bilayer (assuming that the bilayer membrane is a parallel plate capacitor) as defined by eq 3; our data suggests that α_5_β_1_ integrin containing membranes have a greater thickness than pristine membranes.
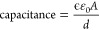
3where ϵ (=2.1) is the relative dielectric constant, ε_0_ (=8.854 × 10^–12^ F/m), *d* is the thickness of membrane (distance between plates), and *A* is the area of the plates [electrode onto which dielectric (here MSLB) is spanned].^44^

Quantitative evaluation of changes to membrane electrical resistance and capacitance properties was achieved by fitting the EIS data to the ECM, as shown in Figure 2G. We previously reported this heuristic approach, applied to the MSLB model.^61,62^ Upon fitting the EIS data (solid lines, Figure 2E,F), representative absolute resistance values for SAM-modified cavity arrays without the bilayer, with PC//PC:PA or PC//PC:PA/Int MSLBs were determined to be 0.4 ± 0.15, 7.7 ± 0.5 and 9 ± 0.8 MΩ, respectively. Similarly, the capacitance (*Q*) values of the constant phase element (CPE) were estimated to be 40 ± 3, 3.4 ± 0.2, and 2.5 ± 0.3 μF·s^*m*–1^, respectively. From our fitting, the other components such as electrolyte solution resistance (*R*_el_ = ∼40 ± 10 Ω), stray capacitance (*C*_stray_ = ∼1 nF) due to the electronic connectors, cavity resistance (*R*_cavity_), and cavity CPE (*Q*_cavity_) remained essentially unchanged. The complex capacitance (*Q*) when CPE is used can be expressed as 1/*Q*(jω)^*m*^, where *Q* is analogous to the magnitude of capacitance, ω is the angular frequency (rad/s), and *m* is the homogeneity parameter varying between 0 < *m* < 1. For an ideal capacitor, *m* = 1 and for a pure resistor, *m* = 0. Typically, for MSLBs, *m* = 0.94 ± 0.02, and for cavity *m* ≤ 0.5 ± 0.02, which corresponds to the bilayer membrane when CPE approaches an ideal capacitor, and the array CPE becomes a series RC circuit or Warburg impedance. Although, the capacitance (*C*) can be obtained from the *Q* value using the expression *C*(ω) = *Q*ω^*m*–1^, it is only valid for a specific ω, limited to the specific ECM and thus, was not used in this study. Furthermore, the membrane resistance and CPE stated above without or with α_5_β_1_ integrin containing membranes have not been normalized to the electroactive surface area. This is due to batch-to-batch variations that occur upon microstructuring of surface areas across the ∼1 cm × 1 cm (length × breadth) gold on silicon chips. For example, discontinuities in packing or where PS spheres fail to pack can constitute between 2 and 5% of area. In addition, as the MSLB fabrication step involved a selective SAM modification to the exposed top surface, it was not straightforward to calculate the electroactive area of the electrode using the conventional Randles–Ševčík electrochemical method, as electron-transfer processes would deviate from the reversiblebehavior of a Faradaic electron-transfer redox process. As a result, for lectin binding studies, we reported the relative (Δ) change in membrane resistance and CPE. Nonetheless, the total electroactive area used in our study was 5 ± 0.4 cm^2^ for a bare cavity array without SAM (Figure S2B,C, Supporting Information). This translated to absolute membrane resistance in the presence and absence of integrin of 45 ± 3 and 35 ± 2 MΩ cm^2^, respectively. The respective absolute capacitance (CPE, *Q*_M_) values were thus 0.5 ± 0.07 and 0.68 ± 0.03 μF·s^*m*–1^ cm^–2^, respectively. The respective absolute capacitance (*C*_M_) at a fixed frequency (*f*) of 5 Hz were thus 0.55 ± 0.07 and 0.41 ± 0.03 μF·cm^–2^, respectively, which agree well with the literature capacitance values of a bilayer membrane.^44,47,66−68^

The non-Faradaic EIS signal after the formation of MSLBs comprised of PC//PC:PA with or without α_5_β_1_ integrin was monitored to evaluate membrane stability. When MSLBs were initially placed in contact with PBS buffer, we observed an initial increase in resistance (Δ*R*_M_ = *R*_M_^time=*t*^ – *R*_M_^time=0^) that took 0–1.5 h to equilibrate. Following equilibration, the EIS signal remained stable over a prolonged window of 24 h (Figure 2H). This window of stability was well beyond our experimental time (5–6 h) for the lectin binding studies (vide infra). To be on the safe side, we always allowed membranes to equilibrate for 2 h prior to measurement.

### Electrochemical Characterization of Gal3 Binding to α_5_β_1_ Integrin-Containing MSLBs

EIS was used as a highly sensitive, label-free method to study the influence of Gal3 on the membrane phase or packing. Following measurement of the impedance of the PC//PC:PA/Int membrane, WTGal3 was incrementally added to the contacting solution. After each WTGal3 addition, an incubation time of ∼30 min was applied before EIS was recorded. The relative changes to membrane resistance and capacitance were extracted by fitting the EIS data to the ECM described above. In each instance, the absolute resistance of the protein-reconstituted membrane was evaluated, to ensure it conformed to the expected *R*_M_ and *Q*_M_ values of stable lipid bilayers, as described above and previously.^61^ As discussed , the relative changes in membrane resistance (Δ*R* = *R*_M_^0^ – *R*_M_^lectin^) and capacitance (Δ*Q* = *Q*_M_^0^ – *Q*_M_^lectin^) were determined, where *R*_M_^0^ and *Q*_M_^0^ represent, respectively, the absolute resistance and capacitance of pristine membranes, that is, in the absence of lectin, and *R*_M_^lectin^ and *Q*_M_^lectin^ the respective values when lectin was present in the contacting solution.

**Figure 3:**
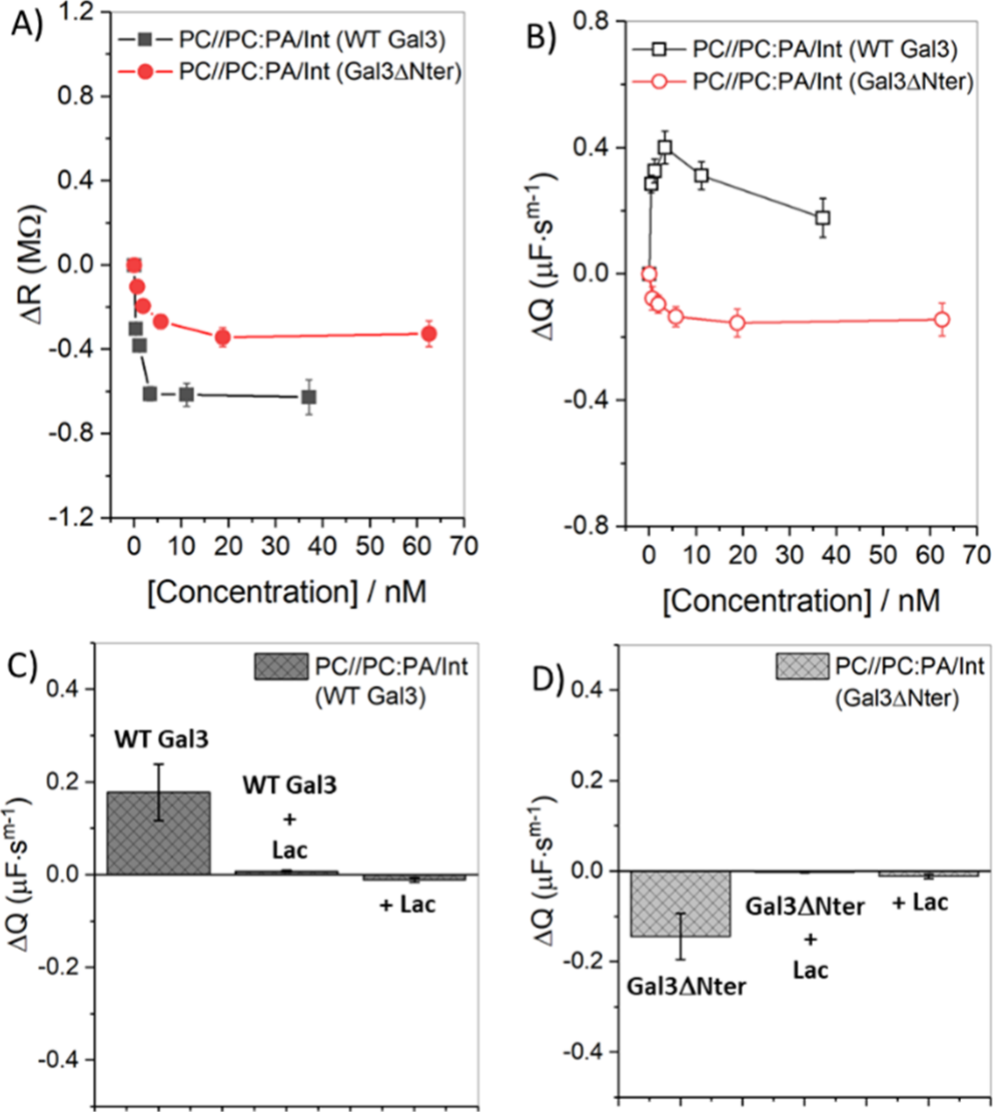
EIS characterization of integrin-containing membranes upon binding of WTGal3 or Gal3ΔNter. (A,B) Relative changes in (A) resistance, Δ*R* (filled symbol), and (B) capacitance, Δ*Q* (open symbols) values obtained upon the addition of different concentrations of WTGal3 (black) or Gal3ΔNter (red) to PC//PC:PA/Int membranes. The solid lines in each panels A and B are shown to guide the eye. Data are means ± SD from triplicate experiments. (C,D) Bar charts showing the change in capacitance values with respect to the pristine PC//PC:PA/Int membrane when 37 nM WTGal3 (C) or 62.5 nM Gal3ΔNter (D) were incubated with α_5_β_1_ integrin-containing membranes in the presence (+Lac, 50 mM) or absence of β-lactose. Lactose fully abolished the WTGal3 or Gal3ΔNter effects on Δ*Q*. In the absence of Gal3, lactose did not affect Δ*Q*. EIS measurements were performed in PBS buffer, frequency ranges were from 0.05 to 10^5^ Hz at 0 V DC bias potential vs Ag/AgCl (1 M KCl) with an AC amplitude of 10 mV at 22 ± 1 °C. The measurement cell was a three-electrode setup where gold cavity/MSLB, Ag/AgCl (1 M KCl), and Pt wire served as working, reference, and counter electrodes, respectively.

Upon binding of WTGal3 to PC//PC:PA/Int MSLBs, the membrane resistance decreased and capacitance increased (Figure 3A,B, black). Although the binding of a Gal3 mutant in which the unstructured N-terminal oligomerization domain was deleted (termed Gal3ΔNter) also reduced the membrane resistance, the magnitude of resistance changes was found to be consistently roughly half of the one that was found or WTGal3. Capacitance values increased with increasing WTGal3 concentration up to 10 nM (Figure 3B, black), whereas a systematic decrease was observed for Gal3ΔNter that saturated at 10 nM (Figure 3B, red).

In the absence of α_5_β_1_ integrin, that is, at PC//PC:PA membranes, WTGal3 elicited a systematic decrease in membrane resistance with increasing concentration (Figure S5A, black, Supporting Information) but the response was weaker compared to PC//PC:PA/Int membranes. The decreased membrane resistance was consistent with the increased diffusivity of the membrane reported in FCS studies vide infra and may be due to changes to membrane packing or nanopore formation induced by WTGal3. WTGal3 also caused a very small (∼0.1 μF·s^*m*–1^) initial increase to capacitance that stabilized at 10 nM (Figure S5B, black, Supporting Information). In contrast, even at concentrations up to 62.5 nM, Gal3ΔNter did not elicit any measurable change to PC//PC:PA membrane resistance (Figure S5A, red, Supporting Information) or capacitance (Figure S5B, red, Supporting Information).

Overall, the data suggest that WTGal3 interacts with the PC//PC:PA membrane in the absence of integrin and increases membrane ion permeability. This may be mediated through electrostatic interactions between negatively charged egg phosphatidic acid (PA) head groups and the CRD of Gal3, which is positively charged at a physiological pH of 7.4,^15^ or via penetration of the partially lipophilic proline-rich N-terminal domain^16^ into the lipid bilayer, possibly inducing some surfactant-like nanoporation.^69^ That Gal3ΔNter did not affect the electrical membrane properties argues in favor of the latter explanation.

The impact of WTGal3 on membrane electrical properties (Figure S5B, black, Supporting Information) was much greater at integrin-containing membranes (Figure 3B, black), which indicated that the glycoprotein strongly favored the recruitment of the lectin. Because these changes were not observed with Gal3ΔNter (see above), we conclude that they were dependent on the oligomerization capacity of WTGal3. We speculate that the hydrophobic proline-rich N-terminal domain-dependent formation of Gal3 oligomers on α_5_β_1_ integrin-perturbed membrane organization, possibly by creating membrane domains and/or pores (see the Conclusions section). WTGal3 has indeed been shown to interact directly with lipid membranes.^69^

Furthermore, at WTGal3 concentrations above 10 nM, membrane resistance stabilized but capacitance gradually decreased suggesting progressive thickening of the membrane (Figure 3B, black). This might have originated from the formation of WTGal3−α_5_β_1_ integrin assemblies in clusters that would have grown laterally into lattices as membrane invagination cannot occur in the MSLB system. Of note, the WTGal3 effect on ion permeability persisted under these conditions as resistance remained at a low plateau level (Figure 3A, black) and capacitance—even if decreasing with the continued addition of WTGal3 above 10 nM—globally also remained above the level that was observed in the absence of the integrin (Figure S5B, black, Supporting Information). We speculate that the permeability and thickening effects occurred concomitantly at WTGal3 concentrations above 10 nM.

The disaccharide β-lactose binds into the core pocket of the CRD on galectins.^13^ It can, therefore, be used as a competitive inhibitor of interactions that depend on this binding pocket. We found that the effects of WTGal3 and of Gal3ΔNter on the capacitance and resistance of α_5_β_1_ integrin-containing membranes were eliminated in the presence of β-lactose (Figures 3C,D and S6, Supporting Information). We confirmed that lactose had no effect on membrane thickness in the absence of Gal3 (Figure 3C,D).

To obtain quantitative empirical insights into the association of WTGal3 and Gal3ΔNter with pristine or α_5_β_1_ integrin-containing membranes, the experimental Δ*Q* data (Figures 3B and S5B, Supporting Information) were fit (Figure S7, Supporting Information) to the empirical Hill–Waud binding model according to eq 4
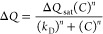
4where Δ*Q* is the change in membrane capacitance, Δ*Q*_sat_ is the absorption capacity or change in capacitance at maximum surface loading that relates to the number of available binding sites, *k*_D_ is the empirical apparent equilibrium dissociation constant, *C* is the concentration of the galectin (WTGal3 or Gal3ΔNter), and *n* (dimensionless) is the Hill coefficient, which reflects the steepness of the slope of the binding curve, often related to cooperativity, where it exists, in protein–receptor binding. *n* < 1 is taken to indicate negative cooperativity, that is, chemically mediated adsorption where binding reduces affinity for further binding events, *n* = 1 reverts the expression to the Langmuir isotherm, where all binding sites are energetically equal, that is, non-cooperative equilibrium binding, and *n* > 1 indicates positive cooperativity, where binding promotes affinity for further binding. From the fitting, a *k*_D_ of 0.2 ± 0.02 nM was obtained when WTGal3 bound to the integrin containing PC//PC:PA membrane versus a *k*_D_ of 0.76 ± 0.04 nM for Gal3ΔNter binding. The fitting parameter values are reported in Table 1.

**Table 1:** Evaluation of Dissociation Binding Constant, *k*_D_, Cooperativity, *n*, and Maximum Saturable Absorption Capacity, Δ*Q*_sat_ of WTGal3 and Gal3ΔNter upon Binding to PC//PC:PA or PC//PC:PA/Int Membranes^a^

lectin	membrane	*k*_D_	Δ*Q*_sat_	*n*
WTGal3	PC//PC:PA/Int	0.2 ± 0.02	0.35 ± 0.05	1.7
WTGal3	PC//PC:PA	0.09 ± 0.02	0.08 ± 0.1	1
Gal3ΔNter	PC//PC:PA/Int	0.76 ± 0.04	–0.15 ± 0.01	0.8
Gal3ΔNter	PC//PC:PA	-	-	-

a *R*^2^ values are 0.95, 0.99, and 0.96 (from top to bottom).

While the comparison between WTGal3 and Gal3ΔNter for the binding to α_5_β_1_ integrin was relevant, *k*_D_ values for binding to membranes with or without α_5_β_1_ integrin could not be directly compared as they reflected on different types of interactions. In the first case, the primary binding site of the Gal3 was α_5_β_1_ integrin glycans, and its effects on membranes needed to be mediated from there. In contrast, in the absence of α_5_β_1_ integrin, the primary binding site was the membrane itself, and the magnitude of EIS signal change was, therefore, expected to be greater. Accordingly, in the presence of integrin, the apparent *k*_D_ for the WTGal3 is approximately 3 times lower than that of Gal3ΔNter (Table 1). While this finding was qualitatively in agreement with the differential effect of both proteins on the capacitance change (Δ*Q*_sat_) (Figure 3B), the amplitude difference between WTGal3 and Gal3ΔNter was much higher for Δ*Q*_sat_ than for *k*_D_. This indicates much larger changes to membrane packing upon binding of WTGal3. This is notably emphasized by the steep slope of the plot (Figure 3A, open black symbol) and the Hill coefficient (*n*) in the presence of integrin (Table 1). The *n* of 1.7 for WTGal3 suggests positive cooperative binding, likely due to Gal3 oligomerization onto the integrin glycoprotein. In contrast, *n* decreased to 0.8 in the case of the Gal3ΔNter, as expected for this oligomerization deficient mutant.

### FCS-Based Characterization of Gal3 Binding to α_5_β_1_ Integrin-Reconstituted Membranes

To further support the EIS data, fluorescence lifetime-based imaging and FCS measurements were carried out at MSLBs on optically transparent arrays.

The PC//PC:PA/Int MSLB preparation over PDMS-based cavity arrays was the same as for the gold microcavities. PDMS is used rather than gold for FLCS as a low reflectance medium was required for single molecule fluorescence. The pore apertures were larger for fluorescence measurements (diameter ∼ 2 μm) to facilitate a pore-by-pore study within the confocal volume.^70^ The pore array substrates for optical studies were incorporated into microfluidic chambers that permitted introduction of reagents to the MSLB with minimum volume (∼150 μL).^56,57,59,70^ The fluidity of the PC//PC:PA MSLBs was evaluated prior to reconstitution of integrin using a fluorescently labeled lipid, ATTO655-DOPE (0.01 mol %), which was doped only at the outer leaflet of the MSLB during liposome preparation, and ATTO532-DOPE (0.01 mol %), which was doped at the inner leaflet. The diffusion coefficients from both leaflets of PC//PC:PA membranes were estimated by fitting the ACF to the 2D diffusion model of eq 1, and the diffusion coefficient at each leaflet were found to be identical as 6.66 ± 0.38 μm^2^/s with an anomalous factor (α) of 0.94 ± 0.17, indicating Brownian diffusion. The lipid diffusivity at the planar PDMS array (in the absence of underlying aqueous pore cavities) was found to be 2.2 ± 0.3 μm^2^/s. The identical diffusivity of each leaflet with greater diffusivity than bilayer supported over planar regions of the PDMS substrate confirmed we were measuring membranes that were suspended across aqueous filled pores with no underlying frictional interaction from the solid support. All FLCS measurements were acquired from the center of the pore using the bright reflectance from the aqueous filled well as a guide and Z-scanning to locate the bilayer. Our reported diffusivity data are averages of at least 40–50 measurements across at least three different substrates. The diffusion coefficient for the PC//PC:PA MSLBs was considerably lower than simple dioleoylphosphatidylcholine (DOPC) MSLBs, which were previously reported as approximately 10.5 μm^2^/s.^56,59^ However, our values matched diffusion values in lipid bilayer membranes made of eggPC^71^ and thereby the greater viscosity of the natural eggPC:eggPA lipids that were used here.^72^

**Figure 4:**
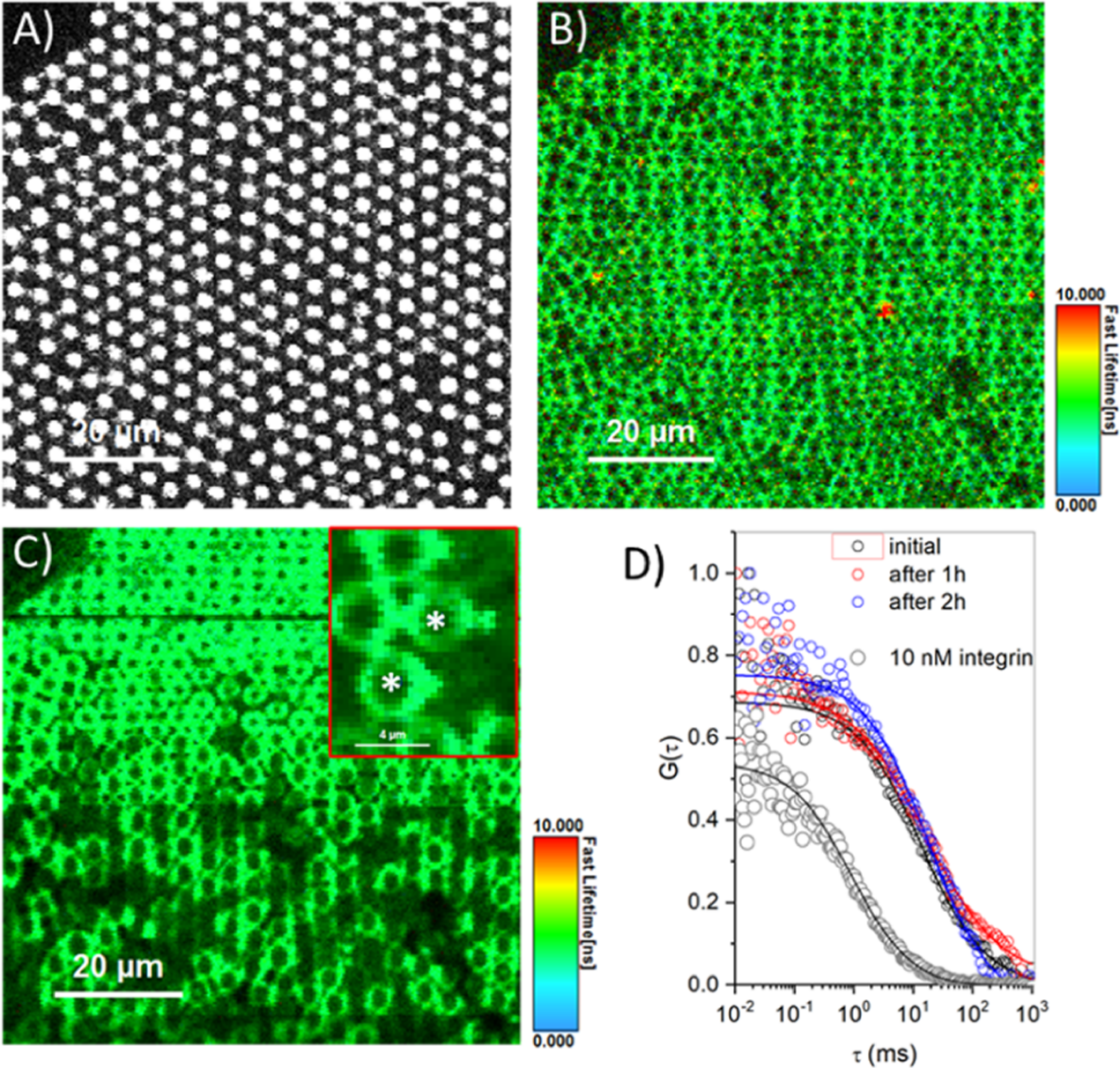
Representative FLIM and FLCS characterization of α_5_β_1_ integrin in MSLBs. (A) Reflectance and (B) FLIM images of ATTO488-labeled α_5_β_1_ integrin reconstituted into a PC//PC:PA MSLBs. Brighter white circles in the reflectance image (panel A) resulted from a refractive index mismatch between the buffer and PDMS. (C) FLIM image of ATTO647-DOPE (0.01 mol %) doped in the lower leaflet. Inset depicts a zoomed-in area, with asterisk “*” highlighting the center of cavity’s spatial regimes, where the membrane was suspended over pores and FCS was acquired from. The scale bars in panels A–C are 20 μm. (D) ACFs for ATTO488-α_5_β_1_ integrin at different time points (black, red and blue) post-reconstitution into MSLBs, along with 10 nM ATTO488-α_5_β_1_ integrin in micellar form diluted in PBS (grey). Solid lines are the fitted data for integrin diffusion across MSLBs and solution using the 2D diffusion model, eq 1, and the pure diffusion model, eq S1 (see the Supporting Information), respectively. The FCS data were collected and averaged from approximately 80–100 points from pores across the substrate. ACF traces showed no changes over 6 h time windows. Both FLCS and FLIM images were taken over PDMS cavity arrays. The substrate was sealed within a microfluidic chamber filled with PBS (pH = 7.4) at 22 ± 1 °C.

To study the diffusion of individual molecules, ATTO488-labeled α_5_β_1_ integrin and Alexa647-labeled WTGal3 and Gal3ΔNter were used. Successful reconstitution of ATTO488-labeled α_5_β_1_ integrin into MSLBs was confirmed by monitoring both FLCS and FLIM at the PDMS platform. Representative FCS autocorrelation data along with FLIM images are shown in Figure 4. The diffusion coefficient of the labeled ATTO488-α_5_β_1_ integrin at the MSLB was measured as 1.99 ± 0.56 μm^2^/s with an α value of 0.86 ± 0.05 in contact with PBS. This value is ∼22× slower when compared to solution diffusivity (46 ± 5 μm^2^/s) of ATTO488-α_5_β_1_ integrin in its micellar form (PBS, 0.2% Triton X-100) at a concentration of 10 nM (Table 2). Compared to the pristine bilayer, the decreased diffusion coefficient of the lipid marker ATTO655-DOPE from 6.66 ± 0.38 to 5.40 ± 0.40 μm^2^/s in the upper leaflet and to 5.85 ± 0.34 μm^2^/s in the lower leaflet indicated that α_5_β_1_ integrin indeed spanned across the bilayer and influenced membrane viscosity at each leaflet. However, lipid diffusion remained Brownian with an α of 0.98 (Table 2). The diffusion of α_5_β_1_ integrin, its molecular brightness, and α value were observed to remain unchanged over a measurement period of 6–8 h, which well exceeded the time window for the lectin binding studies. Interestingly also, the ATTO488-α_5_β_1_ integrin diffusion was impacted by the identity of contacting buffer, whereby when HEPES was used, the average diffusion coefficient was measured at 2.76 ± 0.36 μm^2^/s, instead of 1.99 ± 0.56 μm^2^/s in PBS. However, α was unchanged. Such buffer effects have been noted previously for other membranes.^58,73^

**Table 2:** Estimated Diffusion Coefficients and Corresponding Anomalous (α) Parameter of ATTO488-α_5_β_1_ Integrin in Solution and in MSLBs along with Lipid Diffusion in PC//PC:PA MSLBs in the Presence or Absence of Integrin^a^

diffusing fluorophores	*D* (μm^2^/s)	α
ATTO488-α_5_β_1_ in solution	46 ± 5	1.01 ± 0.03
ATTO488-α_5_β_1_ in PC//PC:PA/Int	1.99 ± 0.56	0.86 ± 0.05
ATTO655-DOPE in PC//PC:PA	6.66 ± 0.38	0.94 ± 0.17
ATTO655-DOPE in PC//PC:PA/Int (upper leaflet)	5.40 ± 0.40	0.98 ± 0.10
ATTO655-DOPE in PC//PC:PA/Int (lower leaflet)	5.85 ± 0.34	0.97 ± 0.12

a PC//PC:PA/Int indicates membranes reconstituted with integrin, and PC//PC:PA membranes without integrin. Data from FLCS studies in PBS at pH 7.4. SDs are from triplicate measurements.

The diffusion coefficient obtained for α_5_β_1_ integrin indicated its proper reconstitution into the MSLB, and the values were comparable to previously reported data for reconstituted integrins in GUVs.^74^ Our values also compared quite well with reconstituted platelet integrin αIIbβ_3_ reconstituted into DOPC or into a complex membrane composition at MSLBs, where diffusion coefficients of 3.20 ± 0.59 and 2.80 ± 0.56 μm^2^/s were reported, respectively, with α coefficients of approximately 1 in these matrices.^55,74^ The lower diffusion values obtained here were attributed to the greater viscosity of the eggPC:eggPA mixture and, in particular, to the greater protein loading in the current protocol where in the originating liposomes a LPR of 2700:1 (mol/mol) is expected to be faithfully translated to the MSLB. Moreover, the α was less than one, indicating sub-diffusion of α_5_β_1_ integrin under our conditions, which we attribute to the somewhat crowded protein environment compared to our earlier reports where integrin was much less concentrated and the LPR was approximately 8000:1 (mol/mol).^55,74^

### WTGal3 and Gal3ΔNter Diffusivity Measurements and Its Impact on α_5_β_1_ Integrin and Lipid Diffusivity across MSLBs

**Figure 5:**
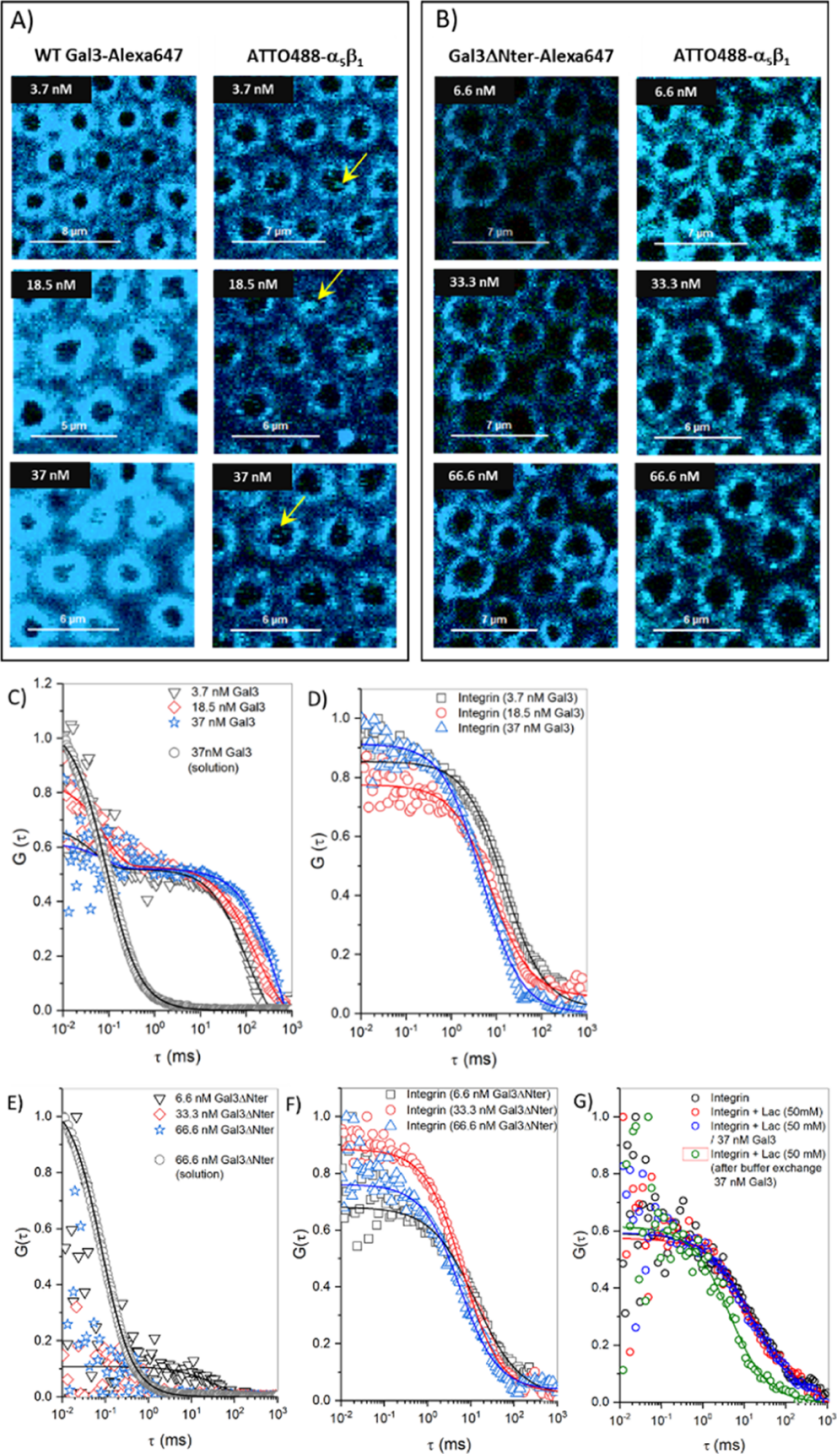
FLIM and FLCS characterization of WTGal3 and Gal3ΔNter upon binding to α_5_β_1_ integrin-containing membranes. FLIM images of (A) WTGal3-Alexa647 (left) and (B) Gal3ΔNter-Alexa647 (left) at varying concentrations upon binding to PC//PC:PA/Int membranes. The corresponding ATTO488-α_5_β_1_ integrin FLIM images are shown to the right. The arrows in panel A indicate integrin clusters in the presence of Gal3. (C,E) ACFs of WTGal3-Alexa647 (C) and Gal3ΔNter-Alexa647 (E) at varying concentrations upon binding to α_5_β_1_ integrin-containing PC//PC:PA membranes. (D,F) ACFs of ATTO488-α_5_β_1_ integrin upon incubation with different concentrations of WTGal3-Alexa647 (D) or Gal3ΔNter-Alexa647 (F). (G) ACFs of α_5_β_1_ integrin-ATTO488 reconstituted into PC//PC:PA membranes (black circle) in the presence of 50 mM β-lactose (red circles), of 37 nM WTGal3 in the presence of 50 mM β-lactose (blue circles), or after exchanging the contact solution of WTGal3 + Lac with fresh 37 nM WTGal3 (olive circles). In all panels (C–G) open symbols represent the experimental data. Solid lines are the corresponding fits using eq 2 except that a pure diffusion model equation (eq S1, Supporting Information) was used to extract the diffusion coefficient values from Alexa-labeled WTGal3 and Gal3ΔNter in solution. All measurements were carried out in PBS buffer of pH 7.4 and at 22 ± 1 °C.

Figure 5A depicts representative FLIM images obtained from the integrin-ATTO488 channel of PC//PC:PA/Int membranes (*right panel*) and from WTGal3-Alexa647 (*left panel*) at different concentrations. The WTGal3-Alexa467 diffusivity in solution was measured at different concentrations between 3.7 and 37 nM and found consistently to be 83 ± 3 μm^2^/s in PBS (Figure 5C, gray circle). From the intensity–time traces throughout 20 independent recordings over 10 s, we did not observe any aggregate intensity spikes or any bleaching, suggesting that at the highest concentration of WTGal3 used here, Gal3 remained monomeric in solution (Figure S8, Supporting Information). Using FLIM and FCS, we then studied the association of Alexa647-labeled WTGal3 or of Gal3ΔNter with PC//PC:PA membranes into which α_5_β_1_ integrin was reconstituted. Based on the intensity of the FLIM signal, the extent of membrane association of WTGal3 at the α_5_β_1_ integrin-containing membrane increased (Figure S9, Supporting Information, for quantification of intensity) in a concentration dependent manner, as depicted in the FLIM images of the Alexa647 channel (Figure 5A, left, top to bottom). The diffusivity of WTGal3 reduced drastically to below 4 μm^2^/s at membranes when compared to the diffusivity in solution (Table 3). On careful analysis across different cavities, we observed two diffusing components: a slow diffusing component, especially at higher concentrations (1.07 and 0.43 μm^2^/s for 18.5 and 37 nM WTGal3, respectively; Figure 5C and Table 3) likely indicating clustering, and a mobile fraction with a diffusion coefficient that reached a plateau at highest protein concentration (3.54 ± 0.28 and 3.59 ± 0.11 μm^2^/s for 18.5 and 37 nM, respectively; Figure 5D and Table 3).

**Table 3:** Averaged Diffusivity Values of α_5_β_1_ Integrin-ATTO488 without and with Varying Concentrations of WTGal3-Alexa647 and WTGal3-Alexa647 Diffusion Values from the Same Experiments and the Corresponding Fits^a^

protein diffusion type at MSLB	*D* (μm^2^/s)	α
integrin before WTGal3	1.99 ± 0.56	0.85 ± 0.26
integrin after 3.7 nM WTGal3	4.31 ± 0.24	1.02 ± 0.26
integrin after 18.5 nM WTGal3	5.01 ± 0.09	0.93 ± 0.20
integrin after 37 nM WTGal3	5.26 ± 0.23	0.92 ± 0.02
3.7 nM WTGal3	2.51 ± 0.28	0.96 ± 0.12
18.5 nM WTGal3	3.54 ± 0.11	0.96 ± 0.08
	1.07 ± 0.15	0.84 ± 0.10
37 nM WTGal3	3.59 ± 0.16	0.92 ± 0.12
	0.43 ± 0.15	0.75 ± 0.18

a The associated anomalous factors are provided. α_5_β_1_ integrin was reconstituted into PC:PA (90:10, w/w) membranes at a LPR of 2700 (mol/mol). Values were collected in a single measurement from 80 to 100 cavity pores with each experiment repeated in triplicate at fresh membrane substrates. The SD were extracted from these measurements.

In the absence of α_5_β_1_ integrin, WTGal3-Alexa647 adsorbed in a concentration-independent manner at PC//PC:PA membranes (Figure S10, Supporting Information), which was consistent with the earlier EIS data. We also observed the emergence of two distinct populations of WTGal3 diffusivity depending on the concentration of WTGal3: a mobile, lower prevalence population that was observed at low Gal3 concentrations (Figure S10F, filled symbol, Supporting Information), and a dominant population observed at high concentrations that showed very low mobility (Figure S10F, open symbol, Supporting Information). The respective diffusivity values were found to be 6.5 ± 0.15, which matched closely to the diffusivity of the lipid probe (6.6 ± 0.38) and 0.1 ± 0.08 μm^2^/s, which may have been due to Gal3 self-aggregation or the preferential association of this population with any gel phases within the membrane. Over the described range of concentrations, unlabeled WTGal3 also modestly influenced the diffusivity of the lipid label ATTO655-DOPE, which increased from 6.6 to 6.9 μm^2^/s (Table S1, Supporting Information). We concluded that membrane packing was somewhat disrupted by WTGal3, which was consistent with the decrease in resistance observed by EIS (Figure S5, Supporting Information). Under identical conditions, much lower association of Gal3ΔNter was observed at the PC//PC:PA membrane (Figure S10E,G, Supporting Information) and no reliable ACF.

Interestingly, when 37 nM of WTGal3-Alexa647 was incubated with PC//PC:PA membranes in the presence of 50 mM β-lactose, we observed very little signal from FLIM imaging, a low ACF signal in FLCS, and a much more modest increase in lipid diffusivity (Figure S11, Supporting Information). Thus, consistent with EIS data, β-lactose seemed to inhibit WTGal3 “non-specific” membrane interaction by preventing its lactose-occupied CRD domain to interact with the membrane. Overall, we speculate that native WTGal3 behaves like an amphiphile: its hydrophobic N-terminus and its positively charged C-terminus may both interact with the lipidic environment, causing the observed decrease in membrane resistance in EIS and altered membrane viscosity.^16^ Our observations are highly consistent with the findings of Lukyanov et al. who reported, on the basis of fluorescence studies, that Gal3 binds to phospholipids and can penetrate liposomal and cell membranes.^69^ As described, the effect is inhibited by lactose and suggests that the sugar induces structural changes in the galectin beyond the CRD. Indeed it has been reported that self-association between Gal3 molecules (which are not glycosylated) is impeded by lactose.^75,76^

While two mobile WTGal3-Alexa647 populations emerged at the membranes in both the presence and absence of α_5_β_1_ integrin, important differences existed. In the absence of integrin, the diffusivity of the mobile population at 6.5 ± 0.15 μm^2^/s (Table S1, Supporting Information) matched closely that of the ATTO655-DOPE lipid at 6.66 ± 0.38 μm^2^/s (Table 2). In contrast, in the presence of α_5_β_1_, the mobile WTGal3-Alexa647 component exhibited a much lower diffusivity of 2.51± 0.28 and 3.54 ± 0.11 μm^2^/s at 3.7 and 18.5 nM of the galectin, respectively (Table 3), which followed the same trend as the diffusion coefficients of the integrin. Similarly, for the immobile population of WTGal3-Alexa647, diffusivity increased from 0.1 ± 0.08 μm^2^/s at the pristine membrane to 0.43 ± 0.15 μm^2^/s when the integrin was present. The fact that Gal3 diffusivity varied in the presence of α_5_β_1_ integrin suggested their co-association in both the slow and fast moving components, where from FCS we observed photobleaching of the integrin (Figure S12A, Supporting Information) when galectin was present, that we attributed to immobilized integrin (see arrow in Figure 5A, right panel). Cross-correlation between integrin and WTGal3-Alexa647 was evident in FCCS studies discussed below for the fast component at MSLBs (Figure S12B, Supporting Information).

**Figure 6:**
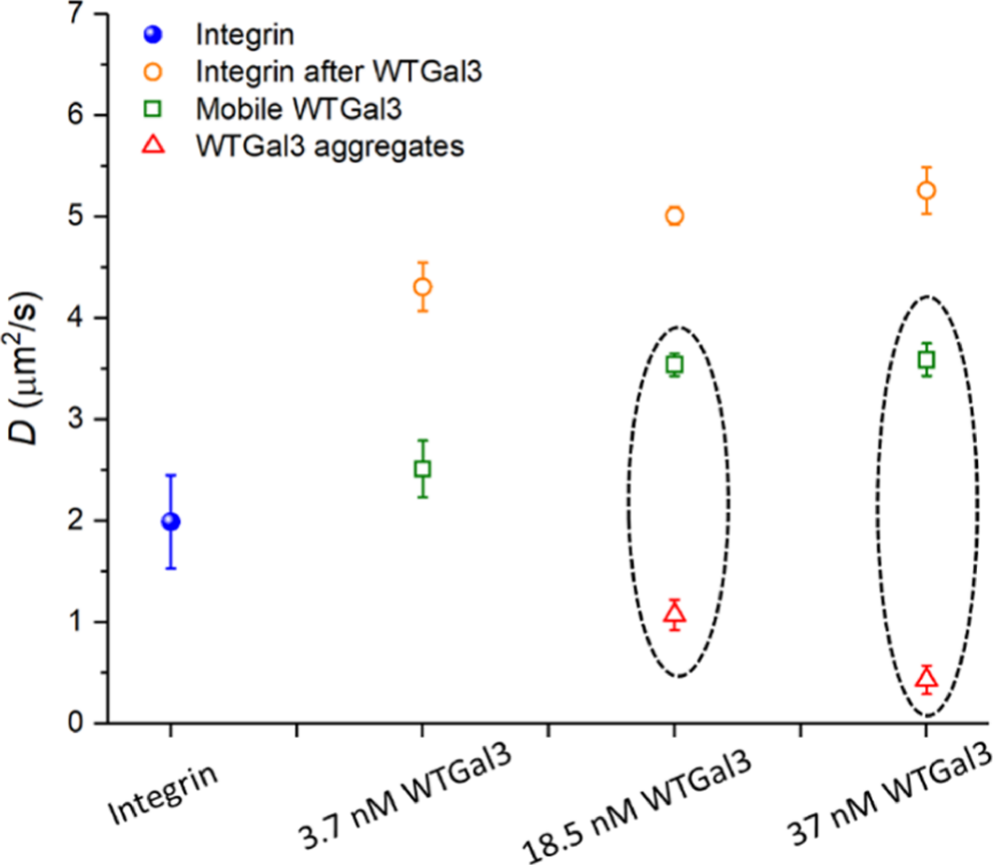
Diffusivity of α_5_β_1_ integrin and WTGal3. Diffusivity of ATTO488-α_5_β_1_ integrin and of WTGal3-Alexa647 at the indicated concentrations of the latter. The blue filled circle shows the diffusion coefficient value for ATTO488-α_5_β_1_ integrin at MSLB before WTGal3 addition. Orange circles are the diffusion coefficient values of ATTO488-α_5_β_1_ integrin following incremental addition of WTGal3-Alexa647 (3.7, 18.5 and 37 nM). Green squares show the fast component of concentration-dependent diffusion coefficient values for WTGal3-Alexa647 after binding to ATTO488-α_5_β_1_ integrin containing PC//PC:PA membranes, and red triangles the ones of the slow diffusing component. Diffusion values in the presence of WTGal3-Alexa647 were obtained following 30 min incubation at the membranes at the indicated concentrations. Dotted oval marks highlight the bimodal diffusivity values of WTGal3-Alexa647.

WTGal3 was observed to increase the diffusivity of α_5_β_1_ integrin in MSLB membranes by at least 2-fold compared to the original diffusion value (Figures 5D and 6, Table 3). This was accompanied by an increase in α to nearly 1 (Table 3), indicating a switch from the sub-diffusion to normal diffusion regime. As described above, the interaction of the WTGal3 with the underlying membrane was observed to decrease its viscosity and this is expected to contribute, at least in part, to this effect. However, both the greater magnitude of the change and alteration in the diffusion regime indicate that other parameters are at play. These may include membrane phase changes or nanopore formation induced by WTGal3 when the integrin is present. However, it is important to note that it is likely that only a subpopulation of integrin remained mobile after galectin addition. α_5_β_1_ integrin tied up in immobile lattices would not have contributed to the FCS signal, except as a background bleach which was evident in the time traces. Such immobilization would have depleted the concentration of mobile α_5_β_1_ integrin in the bilayer. It has been shown previously that increased concentrations of membrane proteins increase membrane viscosity, thereby slowing the diffusion of lipids and of the proteins themselves.^77−79^ This may well explain why we observed an increased diffusion of integrin and an increase in α, as the remaining mobile α_5_β_1_ integrin in the bilayer would have been in a less crowded environment after galectin sequestered a sizable fraction of the protein into immobile networks.

Figure 6 traces the trends in α_5_β_1_ integrin and galectin diffusivities upon incubation with increasing concentrations of WTGal3. α_5_β_1_ integrin and the fast-moving component of WTGal3 followed the same trend toward increased diffusivity when the concentration of WTGal3 was increased. However, the diffusivity values were not identical between integrin and WTGal3. Different explanations may be proposed for this. WTGal3 sub-populations that are not involved in binding to integrin but merely associated with the membrane may have contributed to lowering the mean diffusion values of the fast component of WTGal3 diffusivity. For α_5_β_1_ integrin, it needs to be considered that the protein was randomly oriented in the reconstituted membranes (Figure 1), with 50% of the molecules not exposed to WTGal3. Notwithstanding any immobile integrin, the observed diffusivity values for α_5_β_1_ integrin were, therefore, likely to be a mean of 2 subpopulations of which only one was in contact with WTGal3. At WTGal3 concentrations of 18.5 nM and above, when the slow diffusivity component of the galectin appeared, α_5_β_1_ integrin diffusivity apparently reached a plateau. This behavior could be due to the WTGal3-driven emergence of large α_5_β_1_ integrin clusters. These interpretations are in line with a recent study where upon the addition of WTGal3 to HeLa cells, lateral mobility of β_1_ integrin as well as the size of clusters involving the integrin were increased.^80^

### Oligomerization Capacity of Gal3 Is Essential for Efficient α_5_β_1_ Integrin–Gal3 Complex Formation

Compared to WTGal3, Gal3ΔNter-Alexa647 associated more weakly with ATTO488-α_5_β_1_ integrin containing membranes, as revealed by much weaker signal intensity from the FLIM imaging (Figure 5B, left panel). The solution diffusivity of Gal3ΔNter-Alexa647 was determined as 90 ± 5 μm^2^/s (Figure 5E, grey circle) and the Gal3ΔNter ACF signal was too weak to reliably measure diffusivity from the membranes (Figure 5E, black, red, and blue symbols). Furthermore, in contrast to WTGal3, the diffusion coefficient of ATTO488-α_5_β_1_ integrin was unaffected by incubation with Gal3ΔNter-Alexa647 and remained constant at approximately 1.9 ± 0.2 μm^2^/s over all Gal3ΔNter concentrations that were explored (Figure 5F). That we saw evidence for weak physisorption of Gal3ΔNter in the EIS measurement but not by FCS can be attributed to the fact that in the EIS experiments, the galectin was retained in the contacting solution during measurements, whereas in the microfluidics device used for FCS, blank buffer was exchanged to reduce any background contribution from unbound fluorophores. If the bilayer interface was not exchanged with fresh buffer, the lectin diffusion stayed the same as in solution (Figure 5C,E, grey; Figure S12B, blue, Supporting Information).

Evidence for association of WTGal3 with the mobile integrin fraction (Figure S12A, Supporting Information) was reflected in modest but significant cross-correlation data (Figure S12B, Supporting Information) obtained from spatial FLCS measurements. The cross-correlation signal was weak, presumably because much of the integrin–galectin was tied up in immobile networks, and the FCCS signal reflected residual integrin–WTGal3 complexes that diffused with the same *D* as the integrin. It is also important to remember again that 50% of the integrin was in the “upside down” orientation, which prevented it from binding to WTGal3 and becoming immobilized at the galectin network. The weak FCCS signal could, therefore, be attributed to correctly oriented integrin that remained mobile in the membrane and formed a complex with non-networked galectin.

No such cross-correlation was obtained with Gal3ΔNter-Alexa647 on α_5_β_1_ integrin-ATTO488 containing membranes (data not shown), which was again consistent with a weak binding of Gal3ΔNter to α_5_β_1_ integrin.

Of note, no WTGal3-induced clustering of α_5_β_1_ integrin was observed from intensity time traces obtained from multi-channel-scalers when α_5_β_1_ integrin was in the micellar form (Figure S12C, Supporting Information). In contrast, the diffusivity of α_5_β_1_ integrin was modestly increased in its micellar form upon WTGal3 addition as illustrated from ACFs traces (Figure S12D, Supporting Information), suggesting that Gal3 also oligomerized under these conditions.

In the presence of 50 mM β-lactose (Lac), the increase of ATTO488-α_5_β_1_ integrin diffusivity upon WTGal3-Alexa647 addition was no longer observed (Figure 5G). As a control, we showed that the diffusivity of ATTO488-α_5_β_1_ integrin itself was not influenced by 50 mM β-lactose incubation and remained stable at 1.9 ± 0.7 μm^2^/s (Figure 5G). Furthermore, exchange with blank buffer containing freshly added WTGal3-Alexa647 (37 nM) in the absence of β-lactose caused the diffusivity of α_5_β_1_ integrin-ATTO488 to increase to 5.5 ± 0.8 μm^2^/s (α = 0.98) (olive, Figure 5G).

As with EIS experiments, the FCS data established that α_5_β_1_ integrin was recognized by WTGal3 at the membrane in a glycan-dependent manner. This efficient α_5_β_1_ integrin–Gal3 complex formation and the consecutive measurable membrane electrophysical modulations fully relied on a functional Gal3, where both C-terminal carbohydrate recognition and N-terminal oligomerization domains appeared to be essential features.

## Conclusions

We have successfully reconstituted the physiologically purified transmembrane glycoprotein α_5_β_1_ integrin into a microcavity-suspended lipid bilayer (MSLB) platform of complex lipid composition and applied electrochemical tools and fluorescence microscopy to study α_5_β_1_ integrin–Gal3 complex formation and lateral membrane diffusivity. Use of cavity SLBs with their deep aqueous reservoirs at both membrane interfaces permits native-like lateral fluidity of reconstituted membrane protein and enables versatile multimodal interrogation. This original and sensitive approach allowed us to provide a novel investigative angle to understand the specific functions of the C-terminal glycan binding and the N-terminal oligomerization domains of Gal3, in the presence of a naturally glycosylated cargo protein, α_5_β_1_ integrin from the rat liver.

EIS experiments demonstrated that even in the absence of α_5_β_1_ integrin, WTGal3 associated with membranes comprised of PC//PC:PA, as deduced from membrane resistance decrease (Δ*R*) that showed a systematic and saturable response that followed Langmuir behavior with increasing WTGal3 concentrations. Consistent with EIS data, FCS data revealed that the WTGal3 influenced membrane viscosity by modestly increasing the diffusion coefficient of the lipid marker and reducing its α to below 1.

EIS data also showed that even at concentrations below 10 nM, WTGal3 associated with glycosylated α_5_β_1_ integrin in a positive cooperative manner (*n* = 1.7). The simplest interpretation of this data is that WTGal3 oligomerized on individual α_5_β_1_ integrin heterodimers. This was then followed by a gradual capacitance decreases at WTGal3 concentrations above 10 nM, which indicated significant thickening of the membrane. This was attributed to an initial WTGal3-driven integrin clustering that further switch to a lateral condensation of α_5_β_1_ integrins into lattices, resulting in the observed global thickening of the bilayer. This hypothesis is supported by FLIM imaging, where integrin clusters seemed to reorganize between 3.7 and 18.5 nM of WTGal3 to then increased in size between 18.5 and 37 nM. These changes were not observed with an oligomerization-deficient mutant of Gal3 and Gal3ΔNter and furthermore, they were inhibited in the presence of lactose establishing carbohydrate dependency.

In a natural membrane environment, Gal3 oligomerization on glycoprotein cargoes followed by their locally controlled clustering, would be expected to drive narrow membrane invagination, leading to the formation of tubular endocytic pits, through the GL–Lect mechanism. In contrast, the immobilized population of glycoprotein cargoes would correspond to extended galectin lattices.

FCS measurements confirmed association of the WTGal3 with pristine and integrin-containing membranes and revealed the existence of two Gal3 populations one fast and one slow diffusing that were distinct depending on the presence of α_5_β_1_ integrin in the membrane. In the absence of the integrin, the fast-moving component displayed diffusivity values in the range of those observed for the bulk lipid ATTO532-DOPE (6.5 ± 0.15 vs 6.99 ± 0.12 μm^2^/s respectively), suggesting passive association of the lectin at the pristine membrane. In contrast, diffusivity of the fast component was significantly decreased in α_5_β_1_ integrin-containing membranes when compared to the bulk lipid marker (3.54 ± 0.11 vs 5.40 ± 0.40 μm^2^/s, respectively), which suggested that Gal3 bound to the integrin. Notably, incubation of WTGal3 was observed to increase diffusivity of the integrin at the membrane and also altered the diffusion regime from subdiffusion to Brownian diffusion. However, it was evident from time traces of photobleaching and FLIM imaging that a significant portion of the integrin was immobilized upon WT galectin treatment. The observed increase in diffusion of the mobile α_5_β_1_ integrin at the membrane was attributed to this sequestration of a fraction of the total integrin pool into galectin lattices. This would result in a reduced concentration of mobile integrin and thereby to faster protein diffusion by normal Brownian motion in regions of the membrane that now should have been less crowded.

Moreover, the diffusivity rates of both the mobile α_5_β_1_ integrin fraction and the fast diffusing sub-population of WTGal3 followed the same trend, though exact matching of integrin and galectin diffusion rates was not observed. That a substantial portion of integrin remained mobile after galectin treatment could be ascribed to the orientation of the integrin within the membrane: the inward oriented pool (roughly 50%) was a priori not exposed to the exogenously added Gal3 and therefore was not expected to be influenced by it, creating an asymmetry in lateral integrin diffusivity. The population of integrin that was oriented inward was only influenced by general membrane parameters. This is evident from cross-correlation FCS data which showed that a sub-population of the mobile integrin co-diffused with galectin. In addition, FCS showed integrin bleaching in the presence of WTGal3, which along with FLIM imaging indicated that a significant sub-population of the integrin indeed became immobilized upon exposure to WTGal3.

In summary, we demonstrate that WTGal3 bound in a carbohydrate-dependent manner to α_5_β_1_ integrin in integrin reconstituted into artificial lipid bilayer membranes. EIS and FCS studies indicated that this binding occurred in a cooperative process involving the oligomerization of WTGal3 through its N-terminal domain. Very few biophysical models of integrin–galectin interaction have been reported to date, but with the emerging importance of this interaction across a diverse range of diseases, microcavity-suspended bilayers represent a versatile platform for studying processes such as oligomerization and network formation at the membrane, as they have the compositional versatility and laterally fluidity to facilitate such interactions.

## Supporting Information Available

Fabrication of gold microcavity array electrodes, DLS, EIS, FLIM, AFM, and FCS data (PDF)

## Abbreviations

PC: egg phosphatidylcholine
PA: egg phosphatidic acid
DOPC: dioleoylphosphatidylcholine
LUV: large unilamellar vesicle
EIS: electrochemical impedance spectroscopy
FLIM: fluorescence lifetime imaging
FCS: fluorescence correlation spectroscopy
FLCS: fluorescence lifetime correlation spectroscopy
FLCCS: fluorescence cross-correlation spectroscopy
ACF: auto-correlation function
Int: α_5_β_1_ integrin
WTGal3: wild-type galectin-3
Gal3ΔNter: N-terminal truncated galectin-3
