# Supplementary material for: Galectin-3 Binding to α5β1 Integrin in Pore Suspended Biomembranes
Source: J Phys Chem B. 2022 Nov 22;126(48):10000–17. doi: 10.1021/acs.jpcb.2c05717 (PMC9743206; doi:10.1021/acs.jpcb.2c05717)
Supplement: Supplementary file 1 — jp2c05717_si_001.pdf [file jp2c05717_si_001.pdf]

Supplementary Information for

Galectin-3 Binding to α5β1 Integrin in Pore Suspended Biomembranes

Nirod Kumar Sarangi[1], Massiullah Shafaq-Zadah[2], Guilherme B. Berselli[1], Jack Robinson[1], Estelle Dransart[2], Aurélie Di Cicco[3], Daniel Lévy[3], Ludger Johannes[2]*, and Tia E. Keyes[1]*

[1]School of Chemical Sciences and National Centre for Sensor Research, Dublin City University, DCU Glasnevin Campus, D09 V209, Dublin 9, Ireland.

[2]Institut Curie, PSL Research University, U1143 INSERM, UMR3666 CNRS, Cellular and Chemical Biology unit, 75248 Paris Cedex 05, France.

[3]Institut Curie, PSL Research University, UMR 168 CNRS, 75248 Paris Cedex 05, France.

* email tia.keyes@dcu.ie and ludger.johannes@curie.fr

Table of contents:

Total number of pages: 16

Total number of figures: 12

Total number of tables: 1

**Details of figures/tables**	**Page no.**
DLS and FLCCS characterization of proteoliposomes (Fig. S1)	S2
Gold electrode and MSLB fabrication (Fig. S2)	S3-S4
AFM images of bare cavity and PC//PC:PA/Int MSLB (Fig. S3)	S5
FLIM image of PC//PC:PA MSLB over gold microcavity array (Fig. S4)	S6
EIS studies of lectin binding to PC//PC:PA (Fig. S5)	S7
Bar plot of relative resistance change (Fig. S6)	S8
Hill-Waud binding model fit (Fig. S7)	S9
Intensity-time trace of WTGal3-Alexa647 (Fig. S8)	S10
Intensity quantification (Fig. S9)	S11
FLIM and FCS characterizatio of PC//PC:PA with lectin (Fig. S10)	S12
FLIM image of 37 nM WT Gal3-Alexa647 with 50 mM Lac (Fig. S11)	S13
Intensity-time trace, FLCS and FLCCS (Fig. S12)	S14
Table S1	S15
3D diffusion model for estimation of D	S16

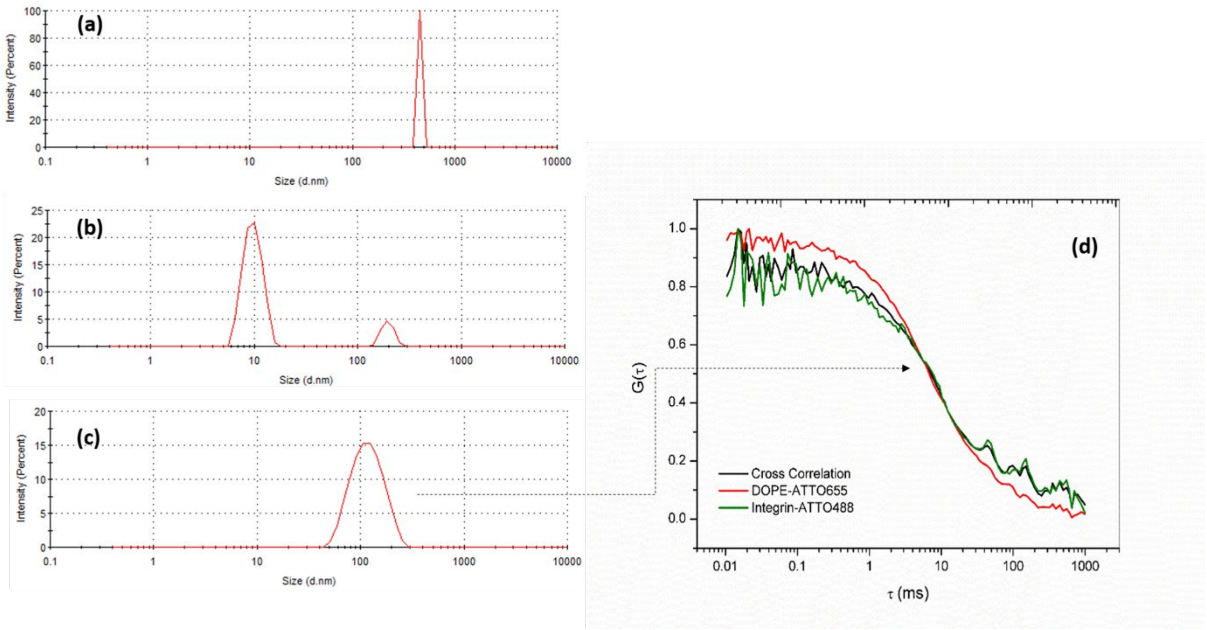

Figure S1. Dynamic Light Scattering (DLS) and Auto correlation functions (ACFs) obtained for Fluorescence Lifetime Cross-Correlation Spectroscopy (FLCCS) of proteoliposomes after reconstituting $\alpha_5\beta_1$ integrin using two independent fluorophores. DLS represents size distribution of (a) liposomes comprised of PC:PA(9:1) with $\alpha_5\beta_1$ integrin at a lipid to protein ratio of 2,700 mol/mol (l/p) after resuspension into HEPES buffer, (b) after destabilization with Triton X-100, and (c) after detergent removal using bio-beads. (d) ACFs obtained from FLCS and FLCCS of proteoliposomes. The red and the green lines represent the ACFs of fluorescent labelled ATTO488-$\alpha_5\beta_1$ integrin and DOPE-ATTO655 (0.01 mol%), respectively, and the black line indicates the cross-correlation between the two dyes.

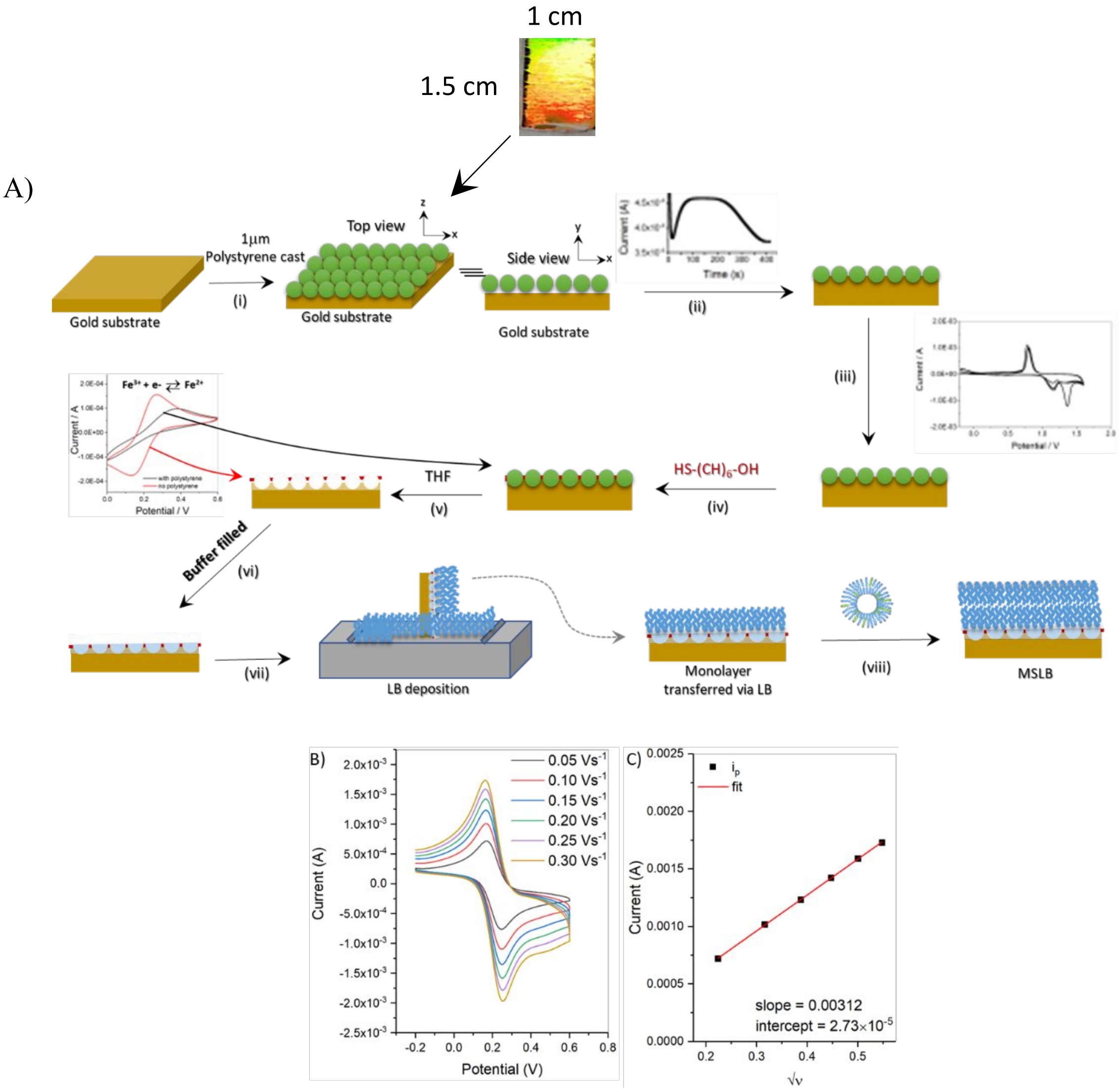

Figure S2. A) Schematic shows the fabrication steps of a gold microcavity array and a microcavity suspended lipid bilayer. Step i: Dropcasting 1 μm diameter aqueous polystyrene beads (1 wt%) onto a flat gold substrate (~ 1 cm × 1.5 cm, *top*) resulted in a hexagonal closed packed array after slow evaporation. Step ii: Submerging a polystyrene-covered gold electrode in gold electroplating solutions resulted in control potential (-0.6 V versus Ag/AgCl) gold deposition. At 400 s, gold deposition covered up to half of the polystyrene sphere diameter in between the interstitial space of the hexagonally packed polystyrene array, as measured by the shape of an I-t curve. Step iii: In a 50 mM H_2SO_4 aqueous solution, the gold top interstitial space was electrochemically cleaned by sweeping the potential from -0.2 V to 1.6 V for three cycles. The cleaning step removed the oxide layer and improved self-assembled monolayer (SAM) formation. Step iv: To produce SAM, the electode was immerged overnight in an ethanolic solution containing 1 mM 6-mercapto hexanol. Step v: Polystyrene beads were removed with tetrahydrofuran (THF), leaving empty cavities with SAM positioned at the top interstitial space. This step was verified successful using cyclic voltammetry of 1 mM Fe^{2+}/Fe^{3+} redox probe in 0.1 M KCl. The characteristic oxidation/reduction behaviour of the probe at the electrodes with (black) and without (red) the polystyrene template are shown. The oxidation/reduction process was switched OFF due to

the insulating properties of intact polystyrene and SAM (black), but it was switched ON when polystyrene was removed. In a CV (red), the peak to peak separation of the SAM covered cavity array was 142 mV, indicating a quasi-reversible electron transfer process confirming SAM integrity. Step vi: The buffer was filled into the SAM covered cavity array electrode. Step vii: The electrode was subjected to Langmuir-Blodgett (LB) lipid monolayer deposition (Y-type), resulting in a lipid monolayer spanning array. Step viii: The vesicle fusion of liposome/proteoliposome over the monolayer spanned cavity array resulted in a microcavity suspended lipid bilayer (MSLB). B) Representative cyclic voltammogram at different scan rates (0.05, 0.1, 0.15, 0.2 and 0.3 Vs^{-1}) performed on bare gold microcavity arrays in a solution containing 1 mM $K_3[Fe(CN)_6]^{3-}$, 1 mM $K_3[Fe(CN)_6]^{4-}$ and 0.1 M KCl in 0.01M PBS. C) Peak current (i_p) versus square root of scan rate (ν) with linear fit, from which the electroactive area was estimated using Randles–Ševčík equation. A three-electrode setup was employed for all CV and I-t measurements, with gold electrodes as working electrodes, Ag/AgCl (1 M KCl) as reference electrode, and Pt wire as counter electrode.

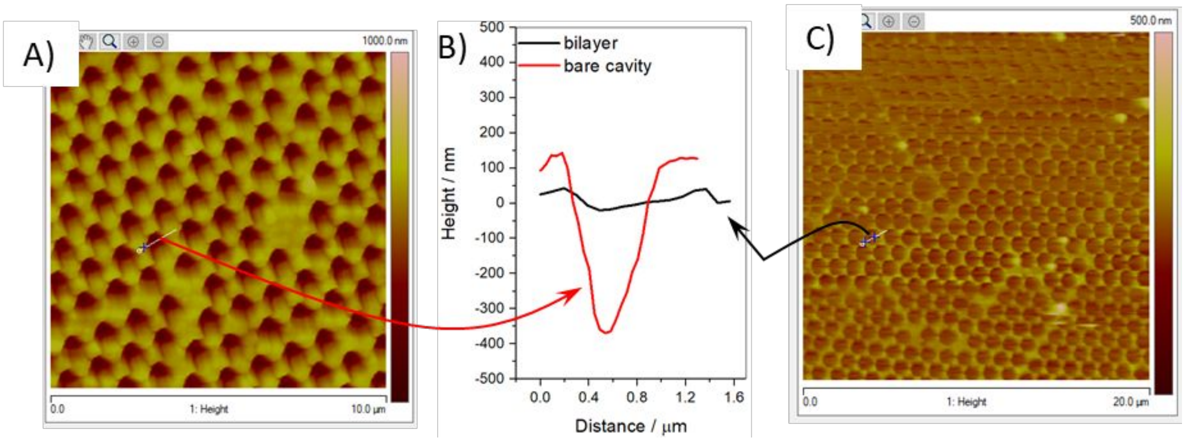

Figure S3. Topographic atomic force microscopy images of A) bare 1 µm cavity and C) cavity spanned with PC//PC:PA/Int membrane. B) Line profile analyses from the regions of interest marked in the respective images by a solid line from which height versus distance plots were obtained as shown in the curved arrow. All images were taken in buffer medium by using tapping-AFM mode. The image was taken under liquid with a scan rate of 0.3 Hz using Veeco Bioscope II system (Nanotec House, Cambridge) coupled with Zeiss Axiovert inverted optical microscope IX70 with silicon nitride cantilevers PNP-TR-20 (NANO WORLD).

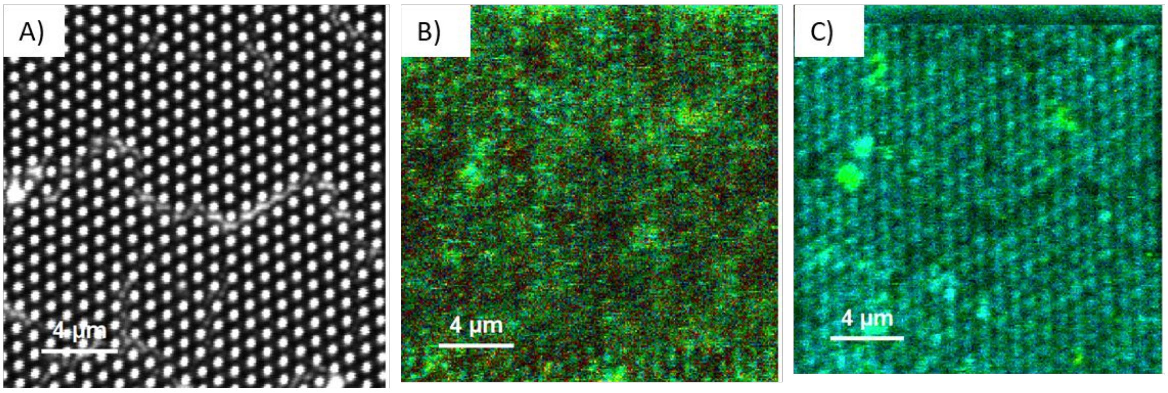

Figure S4. A) Reflectance image showing the buffer filled cavities (white circular feature) over which a PC//PC:PA MSLB was assembled. B) FLIM image of the lower PC leaflet from (A) stained with ATTO532-DOPE (0.01mol%). C) FLIM image of the upper PC:PA(90:10) leaflet stained with ATTO655-DOPE (0.01 mol%). Scale bar are 4 μm.

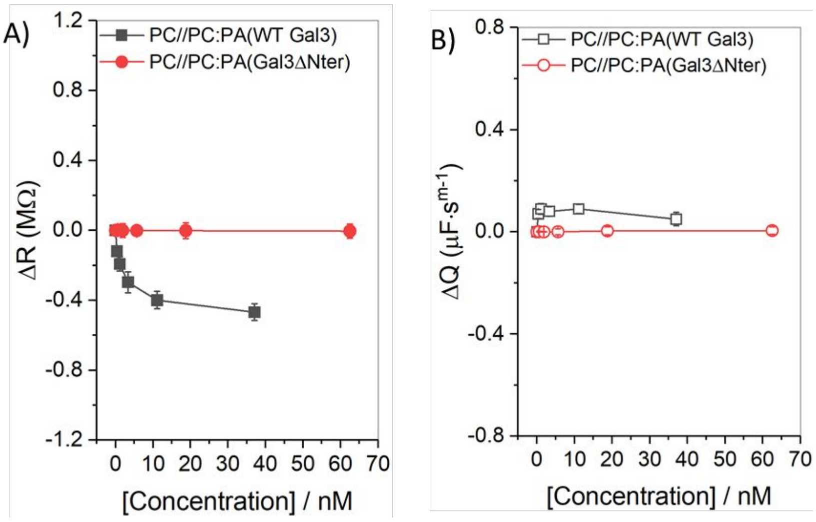

Figure S5. *EIS characterization of pristine PC//PC:PA membranes upon binding of WTGal3 or Gal3ΔNter*. (A,B) Relative changes in (A) resistance, ΔR (filled symbol), and (B) capacitance, ΔQ (open symbols) values obtained upon addition of different concentrations of WTGal3 (black) or Gal3ΔNter (red) to PC//PC:PA membranes.

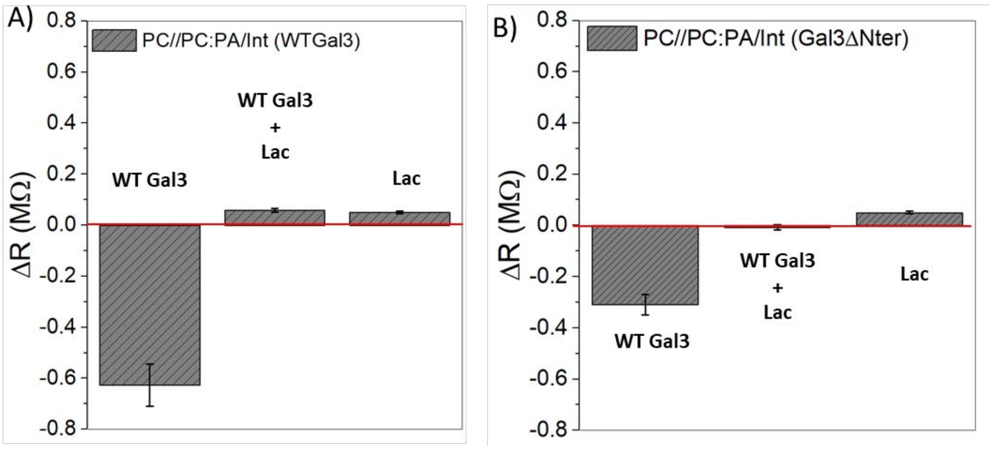

Figure S6. Bar chart showing the relative change in resistance values w.r.t the PC//PC:PA/Int (red horizontal line) membrane when **A**) WTGal3 and **B**) Gal3ΔNter was incubated in the absence or in the presence of β-lactose. Both panel **A,B** also included a control, i.e., β-lactose that was added to PC//PC:PA/Int membrane in the absence of WTGal3 (Lac). The concentrations of WTGal3, Gal3ΔNter and β-lactose were 37 nM, 62.5 and 50 mM respectively. EIS measurements were performed in 0.01 M PBS buffer within frequency ranges between 0.05 Hz to 10^5 Hz at 0 V DC bias potential vs Ag/AgCl (1 M KCl) with an AC amplitude of 10 mV at 22±1 °C. A three-electrode set-up where gold cavity/MSLB, Ag/AgCl (1 M KCl) and Pt wire served as working, reference, and counter electrode, respectively.

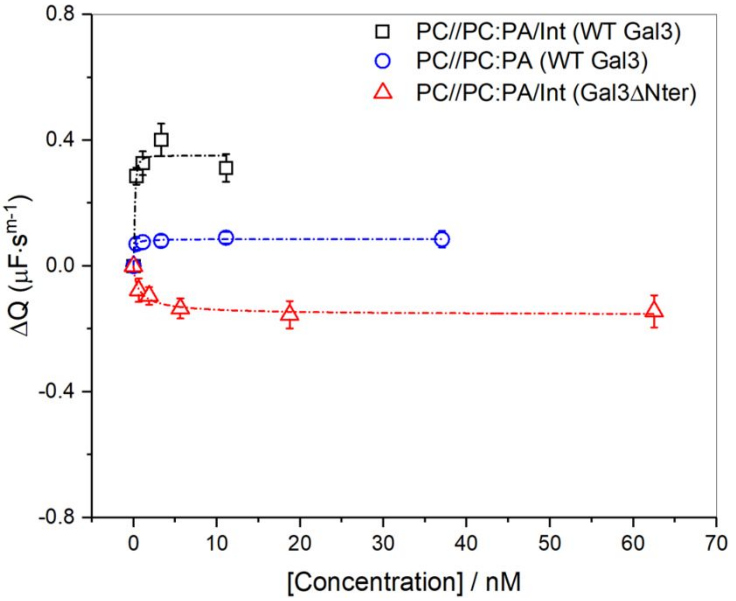

Figure S7. Hill-Waud binding model fit (dotted line) to the relative change in capacitance data of PC//PC:PA and PC//PC:PA/Int membranes upon interaction with WT Gal3 or Gal3ΔNter. Note, since the capacitance changes for PC//PC:PA/Int upon WT Gal3 binding is a biphasic behaviour (see main test), the data point which deviates from saturable binding behaviour (at 37 nM) are not included during Hill-Waud model fit.

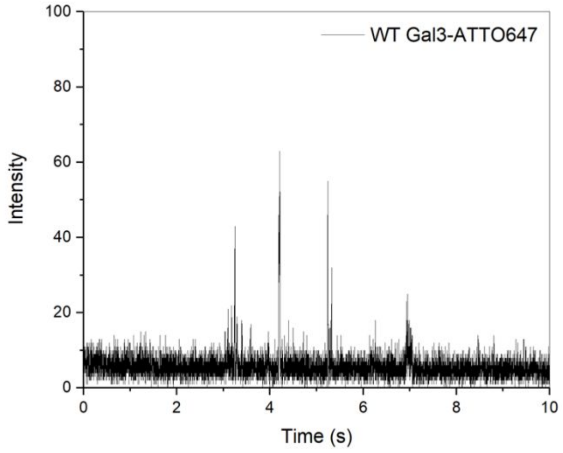

Figure S8. Intensity-time trace of 37 nM WTGal3-ATTO647 diffusing in PBS solution.

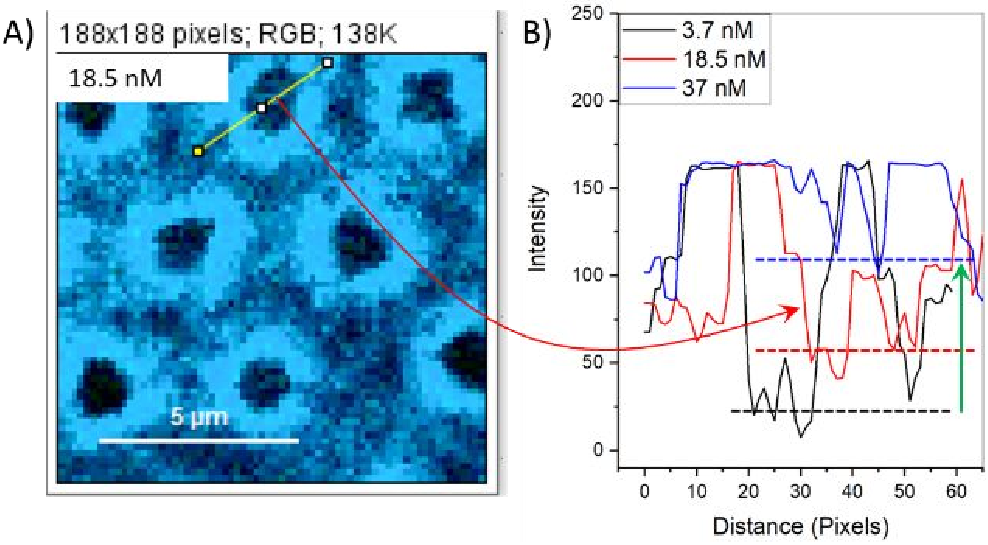

Figure S9. The fluorescence lifetime intensity of WTGal3-Alexa647 after binding to the PC//PC :PA/Int membrane was quantified using ImageJ (v.1.53k). A) In the FLIM image obtained for 18.5 nM (also see Fig. 5A), a representative region of interest (ROI) was drawn by a solid line (yellow), from which intensity counts were taken (red curved arrow). B) Intensity versus distance plots derived from the ROI for increasing concentrations of WTGal3-Alexa647 (black: 3.7 nM, red:18.5, blue: 37 nm). The maximal intensity of WTGal3-Alexa647 obtained from the cavity spanned MSLB regimes was depicted by horizontal dotted lines in panel B, which increased from low to high concentrations, as indicated by the green upward pointing arrow.

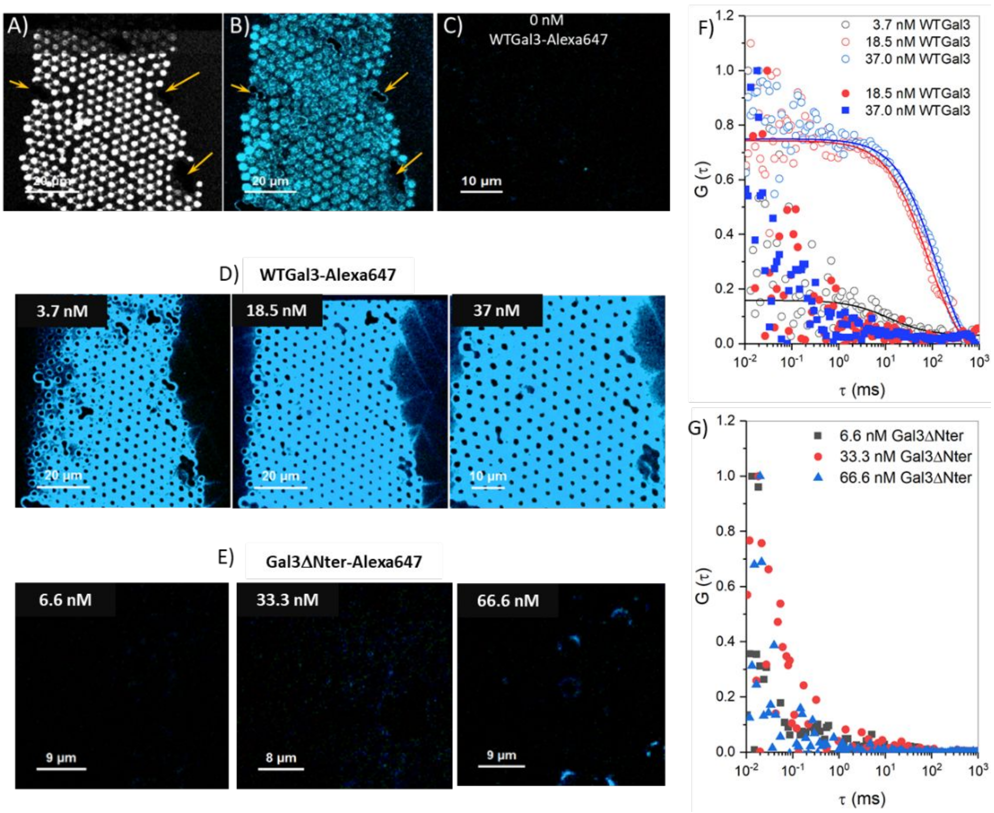

Figure S10. *FLIM and FLCS characterization of pristine membranes upon binding of WTGal3 or Gal3ΔNter.* (**A**) Reflectance and (**B**) FLIM images of pristine PC//PC:PA membranes labelled with ATTO532-DOPE (0.01 mol %). (**C**) FLIM image from the emission channel corresponding to the galectin label, i.e., at 647 nm, when no WTGal3-Alexa647 (0 nM) was added to PC//PC:PA membranes. Arrows in panel (A) and (B) show cavities that were not filled with buffer and where no bilayers were formed. (**D**) and (**E**) represent FLIM images of Alexa647-labelled WTGal3 and Gal3ΔNter, respectively, at varying concentrations upon binding to PC//PC:PA membranes. (**F**) ACFs showing the diffusion of WTGal3-Alexa647 upon binding to PC//PC:PA membranes at different concentrations. Two regions could be distinguished in the array. Open symbols (black, red and blue) show the experimental ACF for 3.7, 18.5 and 37 nM of WTGal3-Alexa647, where extensive aggregation caused slow diffusion. The filled circles represent the experimental ACFs from the spatially resolved regimes with 18.5 nM (red) and 37 nM (blue) of WTGal3-Alexa647, where no diffusion could be detected because of the weak association of galectin at the membrane surface. Solid lines are the 2D fit using Eq (1). (**G**) ACFs of Gal3ΔNter-Alexa647 at varying concentrations (square: 6.6 nM, circle: 33.3 nM, triangle: 66.6 nM) upon binding to PC//PC:PA membranes. For each concentration of WTGal3-Alexa647 and Gal3ΔNter-Alexa647, FLIM imaging and FLCS were acquired after lectin incubation for 30 min and washes off the PC//PC:PA membrane with fresh PBS at 22±1 °C.

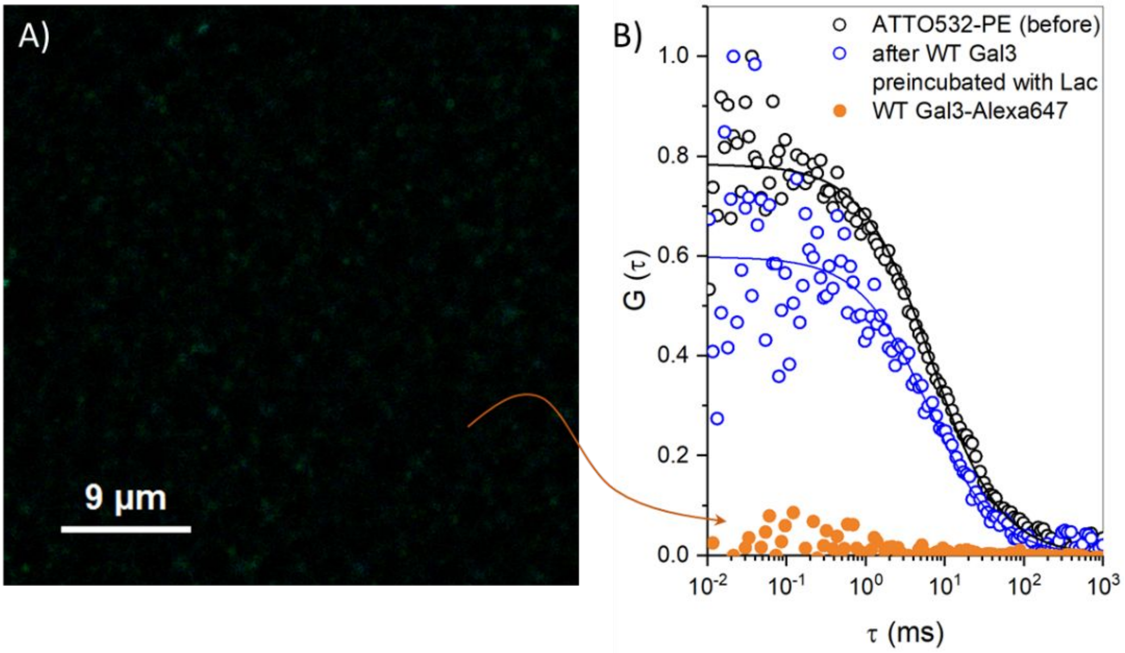

Figure S11. FLIM image of 37 nM WT Gal3-Alexa647 preincubated with 50 mM Lac after binding to PC//PC:PA membranes. No association of the protein to the membrane was observed. B) ACFs of lipid (ATTO532-PE) diffusivity before (open black) and after the addition of 50 mM Lac preincubated WTGal3-Alexa647 (open blue). Filled orange symbols illustrate the ACF of WTGal3-Alexa647 preincubated with Lac that was acquired from the MSLB pores that are shown by the curved arrow. Solid lines are 2D fits.

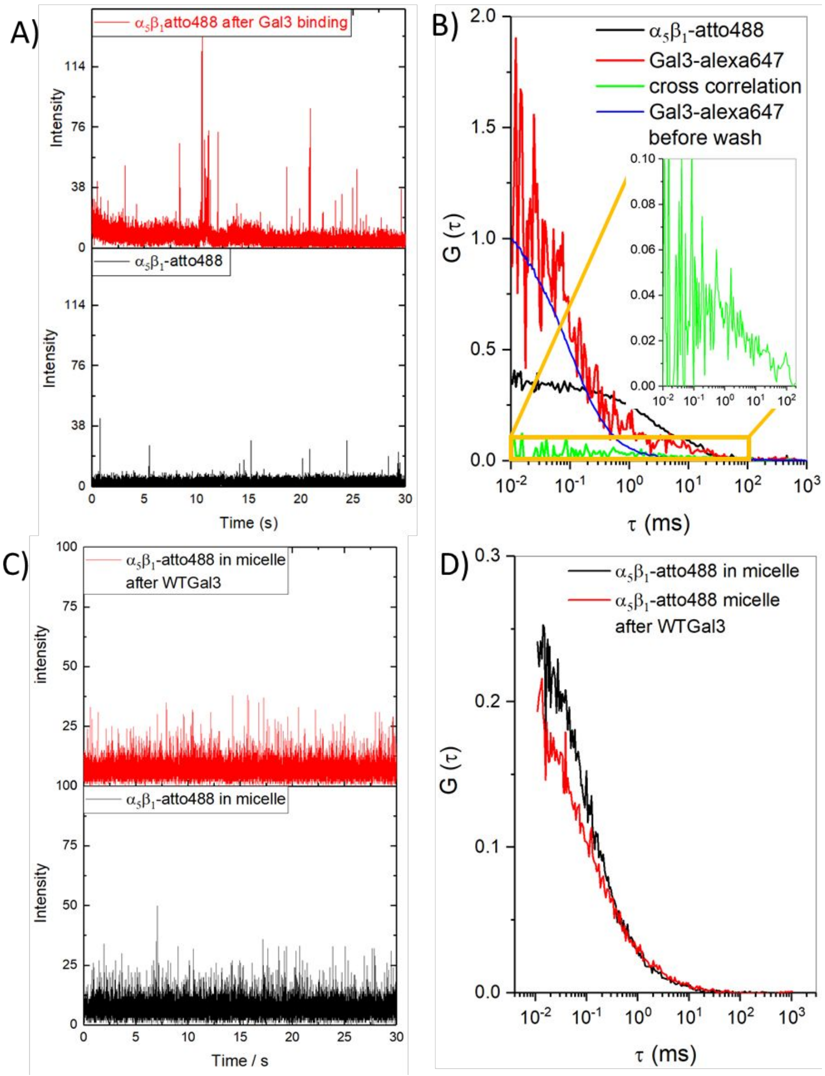

Figure S12. A) Intensity versus time traces obtained from $\alpha_5\beta_1$ integrin-ATTO488 reconstituted in PC//PC:PA/Int membranes before (black) and after incubation with 37 nm WTGal3 (red). From the increased frequency of intensity spikes it is deduced that WTGal3 induced $\alpha_5\beta_1$ integrin clustering. The bleaching of labelled $\alpha_5\beta_1$ integrin at short time points is attributed to the immobilised fraction of the protein. B) ACFs obtained after incubation for 30 min of 37 nM WTGal3-alexa647 with PC//PC:PA/Int membranes upon simultaneous excitation of $\alpha_5\beta_1$ integrin-ATTO488 (black) and WTGal3-Alexa647 (red) and their cross correlation (green), indicating that both diffused together. The inset shows the expanded view of cross-correlation data highlighted in the rectangular orange box. The membrane was washed with fresh PBS buffer before ACF acquisitions in order to remove any unbound WTGal3-Alexa647. Before wash, the ACF of WTGal3-Alexa647 was also included to report on bulk diffusion. C) Intensity versus time traces obtained from α5β1 integrin-ATTO488 in its micellar form before (black) and after (red) 30 min incubation with 37 nM WTGal3. D) ACFs from experiments in (C).

TABLES

Table S1. Estimated diffusion coefficient values of lipids and proteins along with the anomalous factor, α. The SD are from triplicate measurements.

Diffusing fluorophores	D (μm²/s)	α
ATTO532-DOPE in PC//PC:PA	6.6 ± 0.38	0.94±0.17
ATTO532-DOPE in PC//PC:PA after 37 nM WTGal3	6.99 ± 0.12	0.86 ± 0.05
ATTO532-DOPE in PC//PC:PA after 66.6 nM Gal3ΔNter	6.7 ± 0.11	0.97± 0.15
WTGal3-Alexa647 in solution	83 ± 3	1±0.1
WTGal3-Alexa647 in PC//PC:PA (mobile fraction)	6.5 ± 0.15	0.94 ± 0.17
WTGal3-Alexa647 in PC//PC:PA (immobile/aggregates)	0.1± 0.08	0.86± 0.11
Gal3ΔNter-Alexa647 in PC//PC:PA	27 ± 5	0.98 ± 0.10

Estimation of diffusion coefficient using 3D diffusion model.

The autocorrelation function of a freely diffusing fluorescent species in a perfectly three dimension (3D) Gaussian PSF can be expressed by the following equation:

$$G(\tau) = \frac{1}{\langle N \rangle}\left(\frac{1}{1+\frac{\tau}{\tau_D}}\right)\left(\frac{1}{1+\frac{\tau}{\omega^2 \tau_D}}\right)^{1/2} \qquad \text{(S1)}$$

where, $\langle N \rangle$ is the average number of fluorophore molecules in the effective confocal observation volume (V_{eff}), ω, is the structure parameter defined as (σ_z/σ_r), and τ_D is the diffusion time of the fluorophore in the solution/buffer.
